# Ezrin links CFTR to TLR4 signaling to orchestrate anti-bacterial immune response in macrophages

**DOI:** 10.1038/s41598-017-11012-7

**Published:** 2017-09-07

**Authors:** Caterina Di Pietro, Ping-xia Zhang, Timothy K. O’Rourke, Thomas S. Murray, Lin Wang, Clemente J. Britto, Jonathan L. Koff, Diane S. Krause, Marie E. Egan, Emanuela M. Bruscia

**Affiliations:** 1 Department of Pediatrics, Yale University School of Medicine, New Haven, CT USA; 2 Department of Laboratory Medicine, Yale University School of Medicine, New Haven, CT USA; 3 Department of Internal Medicine, Yale University School of Medicine, New Haven, CT USA; 4 Yale Stem Cell Center, Yale University School of Medicine, New Haven, CT USA; 5 Department of Cellular and Molecular Physiology, Yale University School of Medicine, New Haven, CT USA; 6 Quinnipiac University School of Medicine, Hamden, CT USA

## Abstract

Macrophages (MΦs) with mutations in cystic fibrosis transmembrane conductance regulator (CFTR) have blunted induction of PI3K/AKT signaling in response to TLR4 activation, leading to hyperinflammation, a hallmark of cystic fibrosis (CF) disease. Here, we show that Ezrin links CFTR and TLR4 signaling, and is necessary for PI3K/AKT signaling induction in response to MΦ activation. Because PI3K/AKT signaling is critical for immune regulation, Ezrin-deficient MΦs are hyperinflammatory and have impaired *Pseudomonas aeruginosa* phagocytosis, phenocopying CF MΦs. Importantly, we show that activated CF MΦs have reduced protein levels and altered localization of the remaining Ezrin to filopodia that form during activation. In summary, we have described a direct link from CFTR to Ezrin to PI3K/AKT signaling that is disrupted in CF, and thus promotes hyper-inflammation and weakens phagocytosis.

## Introduction

CF is an autosomal recessive genetic disease caused by homozygous mutations at the cystic fibrosis transmembrane conductance regulator (CFTR) gene^1^. Hyper-inflammation is a hallmark of CF’s multi-organ manifestations^2^, contributing to pathology in the lungs, exocrine pancreatic dysfunction^3^, and gastrointestinal tract pathology^4^. The leading cause of death in CF patients is lung pathology, in which production of viscous mucus, hyper-inflammation, and chronic bacterial infection lead to bronchiectasis, small airway obstruction, and, ultimately, respiratory failure. Lung hyper-inflammation, characterized by increased pro-inflammatory cytokines (TNF-α, IL-1β, IL-6 and IL-8) and greater numbers of immune cells, is recognized as a key factor in disease progression^2^.

CFTR encodes for a chloride/bicarbonate membrane protein channel, which is a key transporter involved in maintaining hydration on the mucosal surface of secretory epithelia. Loss of functional CFTR in bronchial epithelial cells leads to airway mucosal dehydration and changes in mucus rheology. Overall, this affects mucociliary clearance, which contributes to chronic bacterial infection. Lower levels of CFTR are also expressed in immune cells, including macrophages (MΦs)^5^. Loss of CFTR in monocytes/MΦs has been associated with increased pro-inflammatory^6–8^ and impaired anti-inflammatory^9–11^ responses, reduced chemotaxis^12^, defective FcR-mediated *P. aeruginosa* internalization^13^ and killing^14, 15^. Thus, together with airway dysfunction, MΦs contribute to CF lung pathology^16^.

We have previously shown that CFTR modulates immune signal transduction in MΦs^6, 16, 17^. Studies of *ex vivo* MΦ cultures isolated from the lungs^18–20^ or peripheral blood (PB)^7, 11, 17, 21^ of CF patients, and animal models suggest that both inherited (lack of CFTR) and acquired factors (CF lung environment) contribute to MΦ dysfunction. As a result, MΦs fail to properly control inflammatory triggers^6, 8, 22^, struggle to resolve inflammation^9–11^, and fail to clear bacteria^13–15, 23^. In addition, MΦs may fail to properly communicate with other immune system cells, thus harming the adaptive immune response^5, 16^.

Over the years, many groups have sought to elucidate how cell-autonomous CFTR dysfunction affects MΦ immune functions. We reported that CFTR modulates the trafficking and signaling of Toll-like receptor 4 (TLR4), an innate immune receptor that senses bacterial lipopolysaccharide (LPS). In the absence of CFTR, TLR4 signaling is dysregulated, leading to enhanced secretion of pro-inflammatory cytokines^6, 17^. One mechanism that we identified to affect TLR4 signaling was the failure of CF MΦs to induce caveolin 1 (CAV1) expression and activation of the heme oxygenase 1/carbon monoxide (HO-1/CO) pathway^10^. Normally this pathway negatively regulates TLR4 signaling, and reestablishes cellular homeostasis^24^. We also showed that CF MΦs have decreased CAV1 expression due to high levels of microRNA-199a-5p (miR-199a-5p), which persists in CF MΦs during TLR signaling, and targets the CAV1 3′-untranslated region (UTR). We showed that high levels of miR-199a-5p in CF MΦs are due to blunted phosphatidylinositol 3-kinase (PI3K)/protein kinase B (AKT) signaling in response to inflammatory triggers^11^. Genetic studies revealed that the AKT1 isoform regulates the miR-199a-5p /CAV1 system in MΦs. Importantly, restoring AKT signaling in CF cells is sufficient to decrease the hyper-inflammatory response to LPS. These findings are consistent with the critical role played by the PI3K-AKT pathway in regulating the immune response^25^, including TLR4 negative regulation in MΦs^26, 27^, MΦ polarization^28^, and innate immune memory acquisition^29^. Here we address the molecular mechanism underlying this dysfunction.

In the search for a mechanism that explains how loss of CFTR leads to blunted PI3K/AKT signaling we hypothesized that Ezrin, a filamentous-actin (F-actin) binding protein and component of the Ezrin-radixin-moesin (ERM) protein family, links CFTR, TLR4, and PI3K/AKT signaling in MΦs. Like other ERM proteins, Ezrin bridges plasma membrane proteins and the underlying cortical actin meshwork, which mediates cell cortex stability in several cell types^30^. It also regulates cellular events that require membrane remodeling, including proliferation, morphogenesis, migration, and adhesion^31, 32^ and modulates plasma membrane signaling transduction^30^. Ezrin has a plasma membrane (PM)-associated FERM domain in the amino terminus, followed by a region with a high α-helical propensity, terminating in a carboxy-terminal domain, known as the C-terminal ERM-association domain (C-ERMAD), which binds the FERM domain (inactive state) or F-actin (active state). Ezrin activation occurs mainly at the plasma membrane^33^. Ezrin binds to phosphatidylinositol 4,5-bisphosphate (PIP2) with its FERM domain. This binding transmits a conformational change through the Ezrin alpha-helical region to weaken the FERM/C-ERMAD association. PIP2-priming of Ezrin permits the lymphocyte-oriented-kinase (LOK) kinase, which has previously been identified as the kinase that phosphorylates Ezrin in lymphocytes^34^, to gain access and to phosphorylate threonine 567 in the C-ERMAD domain (Thr 567). This activation process, unmasks the Ezrin binding sites for F-actin and specific membrane proteins^35^, including the scaffold protein NHERF1 (Na + /H + Exchanger Regulatory Factor), which links Ezrin to CFTR^36, 37^. Because, in addition to the link to CFTR, Ezrin also binds to the C-terminal SH2 domain of PI3K p85, which is critical for PI3K/AKT activation^38^, we hypothesized that Ezrin is a critical link between CFTR and AKT activation.

More specifically, we hypothesized that, in MΦs Ezrin participates in the induction of PI3K/AKT signaling in response to TLR4 activation, and that CFTR is required for this communication. Here, for the first time, we demonstrate that TLR4 signaling in WT MΦs is accompanied by the induction of Ezrin, which is abundantly localized to filopodia that form during activation. Genetic studies reveal that Ezrin is required for sustained PI3K/AKT signaling in response to LPS and that low Ezrin levels impair host defense against *P. aeruginosa*, interfering with the phagocytosis process. Furthermore, we report for the first time that CFTR is necessary for Ezrin localization to the filopodia of activated murine and human MΦs, which means that CF-affected MΦs have reduced Ezrin and blunted PI3K/AKT signaling in response to LPS.

## Results

### Loss of CFTR in MΦs impairs localization of Ezrin to filopodia that form in response to LPS

**Figure 1:**
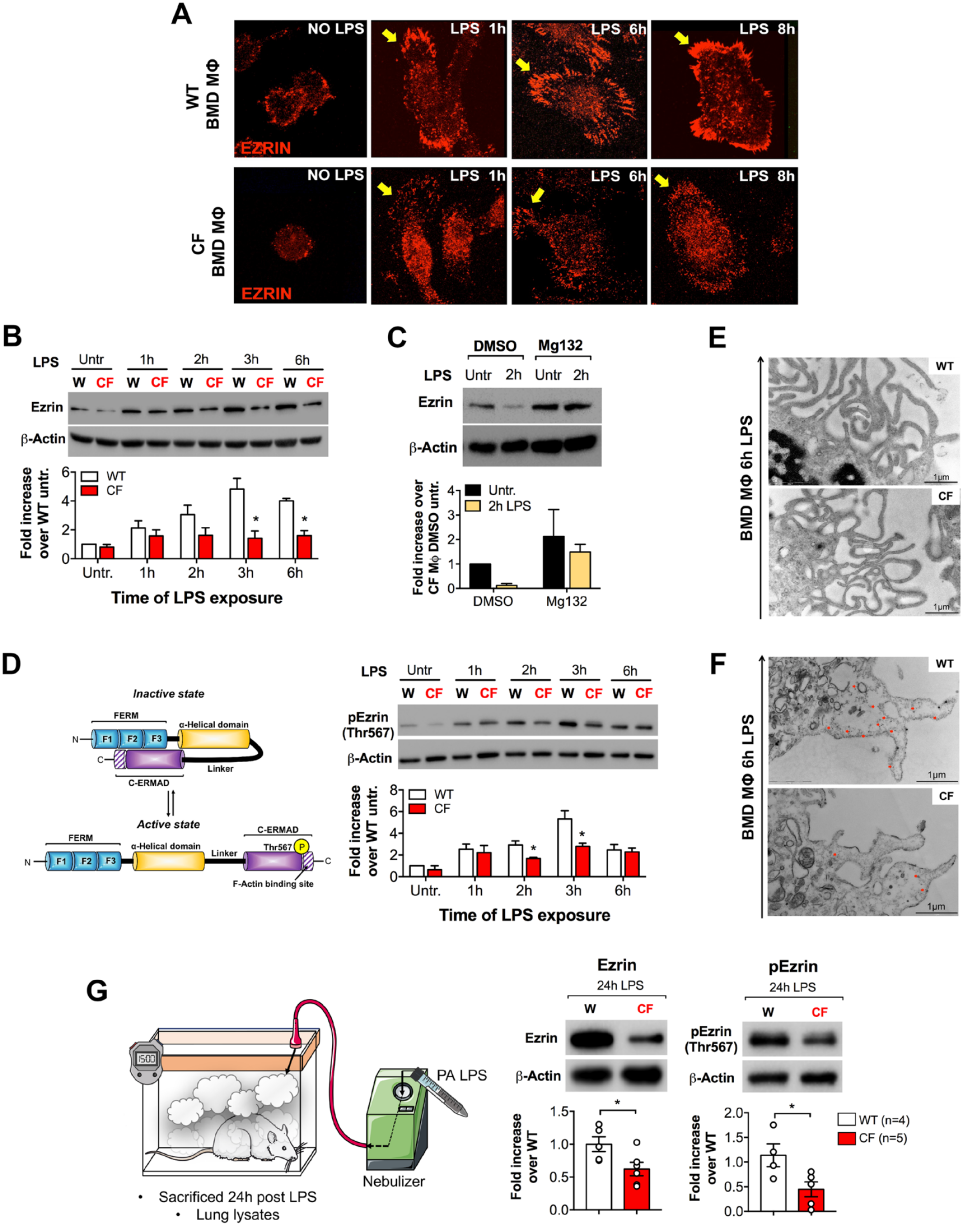
Loss of CFTR in MΦs impairs localization of Ezrin to filopodia that form in response to LPS. (**A**) Immunofluorescence (IF) in murine WT and CF BMD MΦs, untreated or treated with LPS for the times indicated. (**B**) Western blot (WB) and densitometric analysis for total Ezrin (Ezrin). (**C**) WB (upper panel) and densitometric analysis (bottom panel) for total Ezrin (Ezrin) in murine CF MΦs (black bars) and CF MΦs pretreated with the proteasome inhibitor Mg132 (yellow bars), before and after LPS stimulation (2 h). Cells were pre-incubated for 30 min with Mg132 before addition of LPS. DMSO was used as vehicle control. (**D**) Cartoon representation of Ezrin activation (left panel) and WB and densitometric analysis for phosphorylated-Ezrin (p-Ezrin, Threonine 567) in murine WT (white bars) and CF (red bars) BMD MΦs, untreated or treated with LPS for the times indicated (right panel). (**E**) Representative TEM and (**F**) Ezrin immunogold images for WT and CF MΦs treated with LPS (6 h). (**G**) Cartoon representation of the *in vivo* treatment (left panel), WB, and densitometric analysis (right panel) for Ezrin and p-Ezrin in lung lysates from WT and CF mice 24 h after LPS nebulization. For WB, protein fold increase is normalized to β-Actin. Densitometry graphs represent the mean value of three or more (when indicated) biological repeats. Error bars indicate standard deviation. Symbol *indicates a statistically significant difference between the experimental group and control group (*P* < 0.05). Cropped blots are displayed and full-length gels and blots are included in a Supplementary Fig. S8.

We found that WT bone marrow-derived (BMD) MΦs form Ezrin-rich filopodia (Fig. 1A, yellow arrows) and have increased Ezrin levels over baseline in response to LPS (Fig. 1B). In contrast, BMD MΦs from CFTR-deficient mice (hereafter referred to as CF MΦs^39^) have less Ezrin protein in response to LPS, with significantly blunted enrichment of Ezrin at the filopodia, and increased intracellular localization (Fig. 1A,B). CF MΦs pretreated with the proteasome inhibitor Mg132 preserved high Ezrin levels in response to LPS, suggesting that Ezrin is degraded in CF MΦs (Fig. 1C). Indeed, Ezrin mRNA levels in stimulated CF MΦs were not lower than those observed in WT MΦs. On the contrary, CF MΦs expressed compensatory elevated levels of Ezrin in response to LPS (Supplementary Fig. S1A).

LPS exposure also induces phosphorylation of Ezrin’s F-actin binding domain (Thr-567) in WT MΦs, which peaked 3 h after LPS challenge **(**Fig. 1D **)**. In contrast, CF MΦs showed reduced phosphorylation at Thr-567 (Fig. 1D), with the maximum difference in the ratio between active and inactive Ezrin occurring 3 h post stimulation (Supplementary Fig. S1B). This suggested that, in the absence of CFTR, Ezrin might have an altered ability to be activated^31^, which compromises its localization at the forming filopodia^33^ and leads to degradation.

We then investigated whether defective localization of Ezrin to the plasma membrane (PM) during MΦ activation was due to abnormal filopodia formation in CF cells. Transmission electron microscopy (TEM) for WT and CF MΦs revealed no obvious differences in filopodia formation in response to LPS (Fig. 1E and Supplementary Fig. S2). However, immunogold staining confirmed a reduced distribution of Ezrin along the filopodia of activated CF MΦs (Fig. 1F and Supplementary Fig. S3) consistent with the immunofluorescence data (Fig. 1A). Thus, Ezrin is not necessary for filopodia formation in activated MΦs, but its failed localization to these structures might perturb filopodia organization and function.

We further tested Ezrin expression in the lungs of CF mice in response to inhaled LPS. Consistent with the *in vitro* results, CF lung tissue had lower levels of Ezrin expression and activation (Fig. 1G). This difference was also evident in the pEzrin/Ezrin ratio, although this did not reach statistical significance (Supplementary Fig. S1C).

Taken together, these data suggest that Ezrin is part of filopodium structures that form during MΦ activation, and that loss of CFTR alters Ezrin levels, phosphorylation, and cellular distribution in response to TLR4 signaling.

### Ezrin promotes PI3K/AKT signaling downstream of TLR4 activation in MΦs

To test the hypothesis that reduced Ezrin is directly implicated in the blunted PI3K/AKT signaling in LPS-challenged CF MΦs, we generated stable murine MΦ cell lines (J774A.1) that expressed either shRNA against Ezrin or a scramble shRNA-control (shRNA-CTR). We tested four different shRNAs. Two induced up to 70% Ezrin protein inhibition compared to cells infected with retrovirus (RV) controls (Supplementary Fig. S4A). ShRNA-676 stably downregulates Ezrin during LPS challenge (Supplementary Fig. S4B,C). Thus, further experiments were carried out using the shRNA-676 cell lines, hereafter referred to as shRNA-EZR.

**Figure 2:**
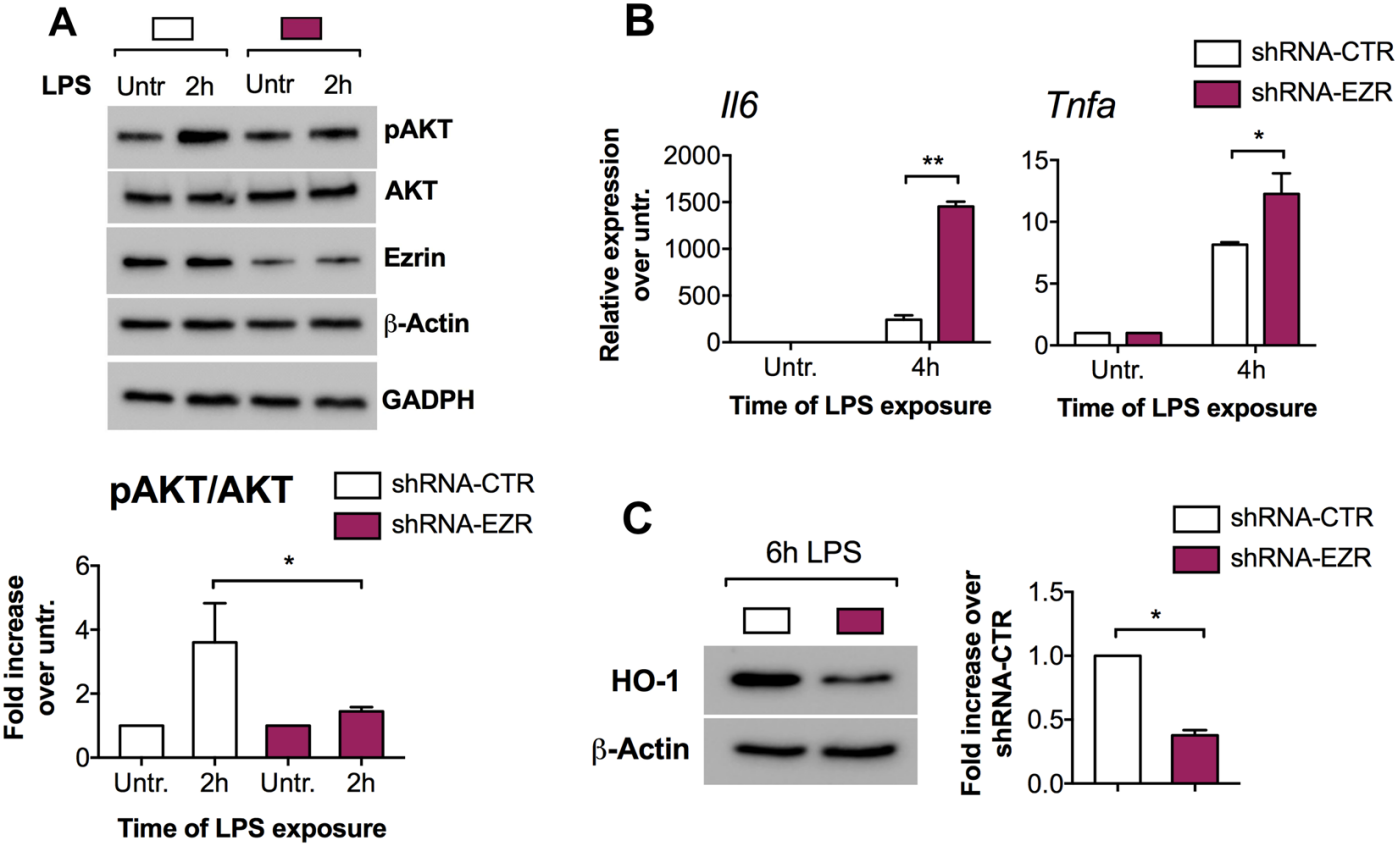
Ezrin promotes PI3K/AKT signaling downstream of TLR4 activation in MΦs. (**A**) WB and densitometric analysis for phospho-AKT (pAKT, Serine 473), total AKT (AKT) and Ezrin in murine J774A.1 cell lines stable transduced with retroviral vector (RV) expressing shRNA against Ezrin (shRNA-EZR, purple bar) or a scramble shRNA control (shRNA-CTR, white bars), untreated or treated with LPS. Bar graph represents the pAKT/AKT ratio. For WB, protein fold increase is normalized to β-Actin. GAPDH is shown as further internal control. (**B**) Quantitative PCR (qPCR) for IL-6 (left) and TNF-α (right) in shRNA-CTR or shRNA-EZR J774A.1 cell lines, untreated or treated with LPS. For qPCR, IL-6/TNF-α expression is normalized to S18. (**C**) WB and densitometric analysis for HO-1 in shRNA-CTR or shRNA-EZR J774A.1 cell lines treated 6 h with LPS. Protein fold increase is normalized to β-Actin. Bar graphs represent the mean value of three biological repeats. Error bars indicate standard deviation. Symbols * indicate a statistically significant difference between the experimental group and control group (*P* < 0.05). Symbol ** indicates a statistically significant difference between the experimental group and control group (*P* < 0.01). Cropped blots are displayed and full-length gels and blots are included in a Supplementary Fig. S8.

After LPS challenge, decreased Ezrin in MΦs impaired AKT phosphorylation but did not affect the total amount of AKT (Fig. 2A), as we previously observed in CF MΦs^11^. Thus, Ezrin is required for efficient PI3K/AKT signaling downstream of TLR4 activation. Furthermore, the J774A.1 shRNA-EZR MΦs challenged with LPS were more pro-inflammatory, as evident by increased expression of IL-6 and TNF-α **(**Fig. 2B **)** than J774A.1 shRNA-CTR, and had reduced HO-1 (anti-inflammatory) protein levels (Fig. 2C), which recapitulates what we previously observed in CF-affected MΦs^11^. Similar results were obtained with cell lines in which Ezrin was downregulated with ShRNA-674 (Supplementary Fig. S4 D–F).

These data suggest that Ezrin is necessary for PI3K/AKT activation, negative regulation of TLR4 signaling, and induction of the anti-inflammatory response in MΦs.

### Ezrin is required for an efficient phagocytic response to P. aeruginosa in MΦs

Several studies have reported that human and murine CF MΦs have impaired phagocytosis with defective FcR-mediated internalization^13^ and killing^14, 15^ of *P. aeruginosa*, a gram-negative bacterium that causes chronic infection in CF lungs. Abnormalities in several cellular pathways, such as autophagy^23^ and phagolysosomal maturation^15^, have been implicated in defective bacterial clearance.

It is well established that PI3K/AKT signaling has a key role on the MΦ’s phagocytic response to *P. aeruginosa* ^40^. In line with these studies, stimulation with *P. aeruginosa* (PAO1 strain) efficiently induces AKT signaling in WT MΦs. However, we demonstrated that this response is defective in CF MΦs exposed to PAO1 (Supplementary Fig. S5A).

**Figure 3:**
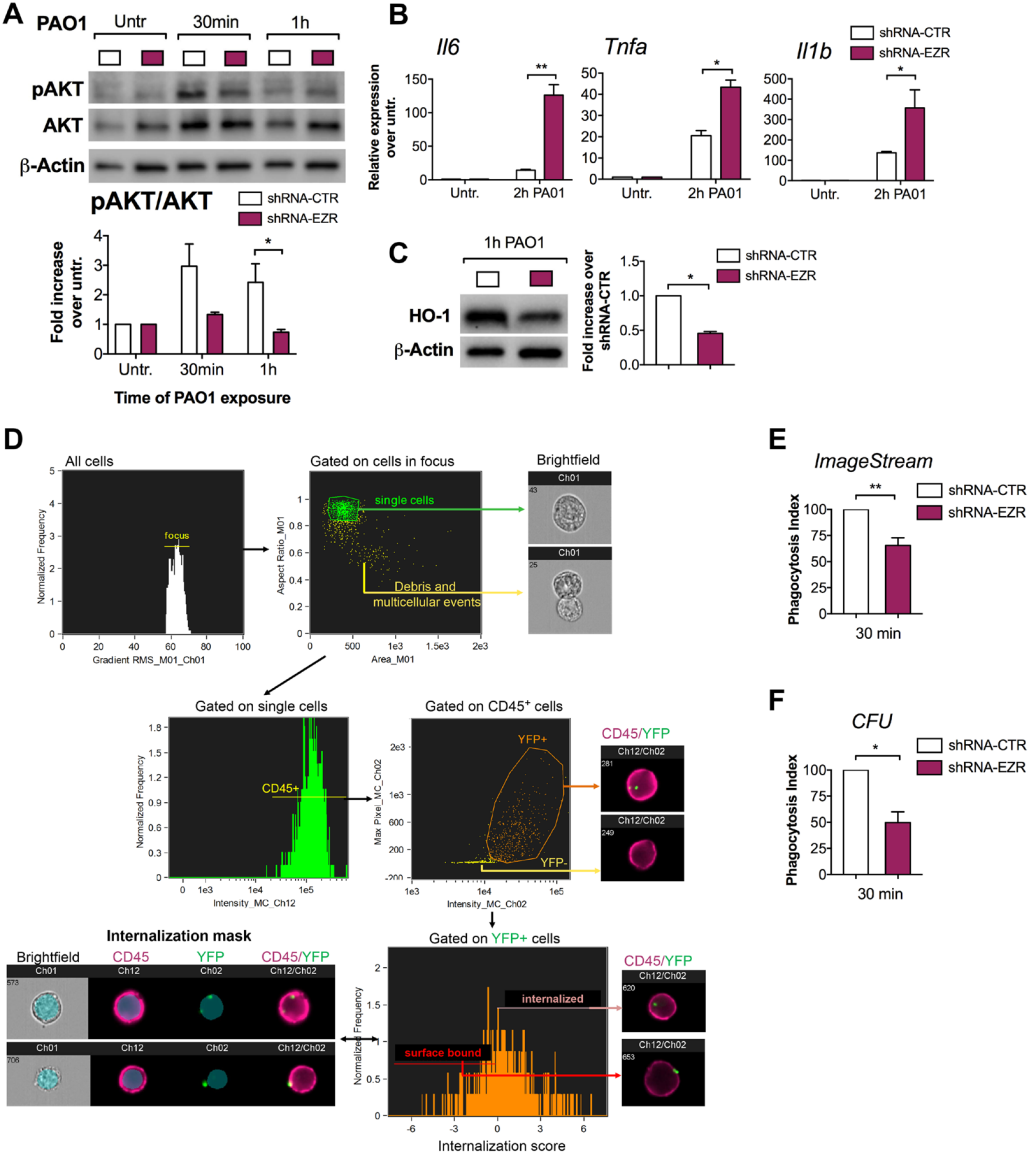
Ezrin is necessary for P. aeruginosa phagocytosis in MΦs. (**A**) WB and densitometric analysis for pAKT and AKT in J774A.1 shRNA-CTR (white bar) and J774A.1 shRNA-EZR (purple bar) cells untreated or treated with PAO1 for the times indicated. Bar graph represents the pAKT/AKT ratio. Protein fold increase is normalized to β-Actin. (**B**) qPCR for IL-6, TNF-α and IL-1β in shRNA-CTR or shRNA-EZR cells, untreated or treated with PAO1. Pro-inflammatory cytokine expression is normalized to S18. (**C**) WB and densitometric analysis for HO-1 in shRNA-CTR or shRNA-EZR treated 1 h with PAO1. Protein fold increase is normalized to β-Actin. (**D**) Representative images showing the gating strategy used to evaluate phagocytosis of PAO1 bacteria (30 min of exposure) by murine J774A.1 cells expressing the shRNA-CTR or shRNA-EZR and stained with CD45. Briefly, a gate was set on cells in best focus (i) and debris and multi-cellular events were excluded to restrict analysis to single cells (ii). CD45 + single cells were gated (iii) and, out of this population, YFP + events were selected (iv). To discriminate between internalized from cell surface-bound bacteria, we created an internalization mask (iv). This internalization mask was then applied to all YFP^+^ cells to calculate the internalization score (vi). Cells receiving an internalization score equal to or greater than 0, were considered to contain intracellular bacteria. For details of the ImageStream gating strategy, please refer to the online supplemental experimental procedures and Supplementary Fig. S5. (**E**) Percentage of J774A.1 shRNA-CTR and shRNA-EZR cells that internalized YFP-PAO1. (**F**) Colony-forming unit (CFU) assay for J774A.1 shRNA-CTR or J774A.1 shRNA-EZR cells exposed to PAO1 for 30 min. Phagocytic index is expressed as the percentage of J774A.1 shRNA-EZR cells that internalized PAO1 (E) or the percentage of PAO1 CFUs (F) over control. For each experiment the following multiplicities of infection (MOIs) bacteria: cells were used: (A–C) 20:1, (D–E) 100:1 and (F) 20:1. For each experiment, data are representative of three experimental biological repeats. Error bars indicate standard deviation. Symbol * indicates a statistically significant difference between the experimental group and control group (*P* < 0.05). Cropped blots are displayed and full-length gels and blots are included in a Supplementary Figure S8.

Given the requirement of Ezrin for an efficient activation of PI3K/AKT pathway in MΦs (Fig. 2), we investigated the effect of Ezrin’s loss on *P. aeruginosa* phagocytosis. J774A.1 shRNA-EZR and shRNA-CTR cells were exposed to PAO1. As observed with LPS **(**Fig. 2 **)**, MΦs with decreased Ezrin stimulated with *P. aeruginosa* had blunted AKT signaling (Fig. 3A and Supplementary Fig. S5B), increased expression of pro-inflammatory cytokines (Fig. 3B), and a reduced anti-inflammatory response (HO-1 protein expression) (Fig. 3C).

To assess phagocytosis, J774A.1 shRNA-EZR and shRNA-CTR cells were exposed to YFP-labeled *P. aeruginosa* (PAO1) for 30 min. The ImageStream imaging-flow platform (Fig. 3D,E and Supplementary Fig. S5C) showed a decreased phagocytosis index in J774A.1 shRNA-EZR cells challenged with *P. aeruginosa* compared to control, establishing that loss of Ezrin impairs *P. aeruginosa* phagocytosis.

Additionally, differences were observed in the number of internalized bacteria/per cell, with J774A.1 shRNA-EZR cells having more cells with single bacteria and fewer with multiple compared to shRNA-CTR cells (Supplementary Fig. S5D), further suggesting less efficient phagocytosis. Defective *P. aeruginosa* internalization in J774A.1 shRNA-EZR was also confirmed by a conventional bacterial CFU assay (Fig. 3F).

In summary, these data show that MΦs with reduced Ezrin have impaired *P. aeruginosa* phagocytosis, which recapitulates CF MΦ dysfunction.

### Primary human MΦs from patients with CF have altered levels and distribution of Ezrin, and blunted PI3K/AKT signaling

**Figure 4:**
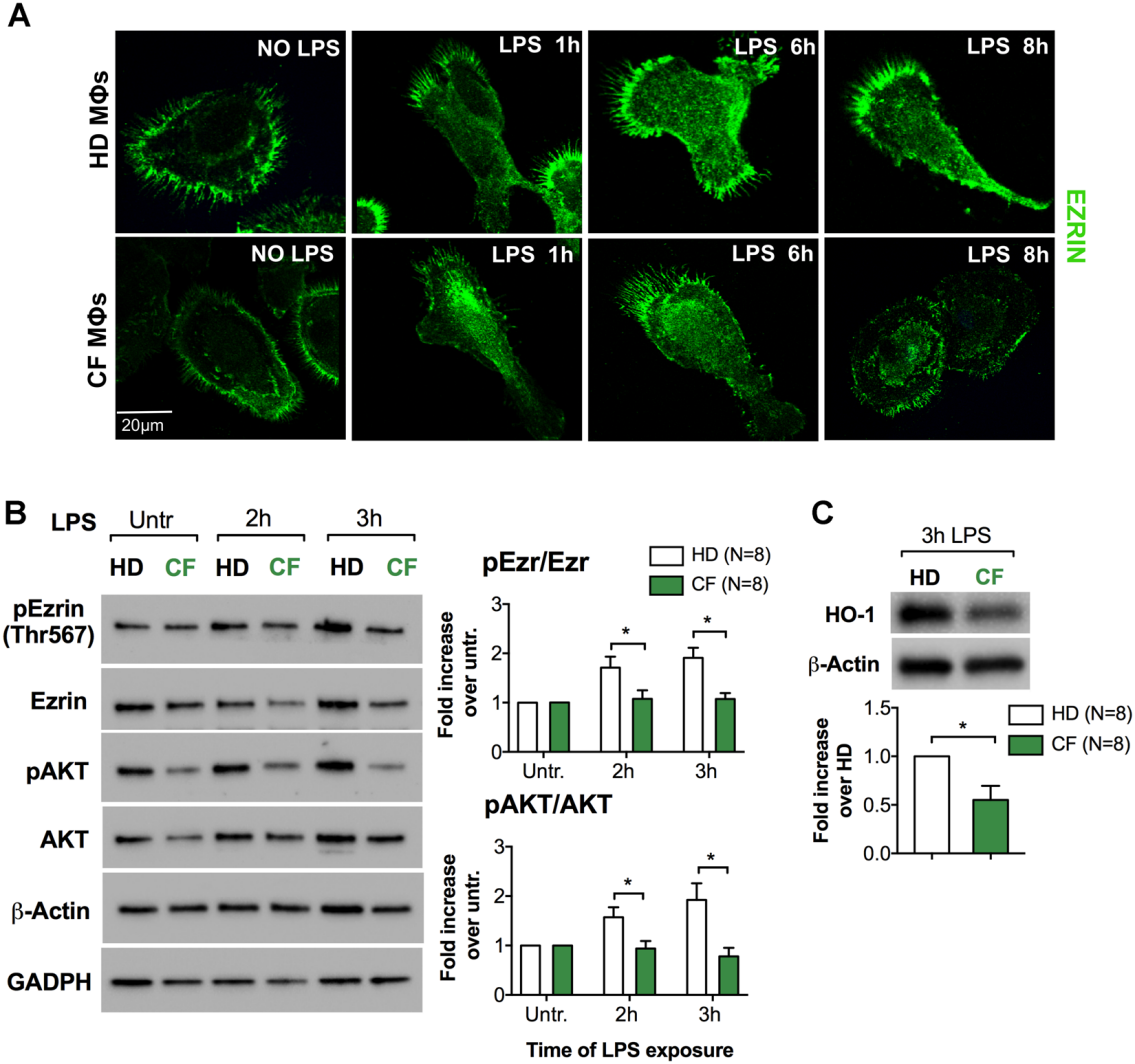
Primary human MΦs from patients with CF have altered levels and distribution of Ezrin, and blunted PI3K/AKT signaling.(**A**) Representative IF for Ezrin in PBD MΦs from healthy donors (HD) and CF subjects (CF). (**B**) WB and densitometric analysis for pEzrin, Ezrin, pAKT and AKT in PBD MΦs from HD (n = 8) and CF (n = 8) subjects untreated or treated with LPS for the times indicated. Protein fold increase is normalized to β-Actin. GAPDH is shown as further internal controls. (**C**) WB and densitometric analysis for HO-1 in PBD MΦs HD and CF patients treated with LPS for 3 h. Data are represented as mean + /− SD of eight HD and eight CF subjects. Error bars indicate standard deviation. Symbols * indicate a statistically significant difference between the experimental group and control group (*P* < 0.05). Cropped blots are displayed and full-length gels and blots are included in a Supplementary Fig. S8.

Previously, we have shown that peripheral blood-derived (PBD) MΦs from CF patients are pro-inflammatory^17^ with altered induction of the miR-199a-5p/CAV-1 pathway and increased expression of several pro-inflammatory cytokines^11^. Here, we investigated the role of Ezrin in PBD MΦs from CF patients (CF PBD MΦs), each of whom had a classic CF phenotype of chronic lung disease and pancreatic insufficiency, and carried at least one copy of the F508del mutation (Supplementary Table S1). We found that CF PBD MΦs had reduced cellular distribution of Ezrin compared to non-CF (healthy donor, HD) controls (Fig. 4A). Though there was donor variability in the kinetics of Ezrin and AKT activation, the heathy donors (HD) always had more robust Ezrin and AKT phosphorylation than the CF patients after LPS stimulation. The data presented in Fig. 4B shows the average of the pEzr/Ezr and pAKT/AKT ratio from 8 HD and 8 CF individuals. Supplementary Figs S6 and S7 shows bar graphs of the β-Actin-normalized total and phosphorylated Ezrin, and total and phosphorylated AKT for each CF patient matched with a healthy donor tested at the same time. Finally, CF PBD MΦs also have lower levels of the anti-inflammatory protein HO-1 in response to LPS when compared to HD controls (Fig. 4C).

## Discussion

The leading cause of death in CF is lung disease, which is characterized by hyper-inflammation and chronic infection, for which *P. aeruginosa* is a significant pathogen^41^. Hyper-inflammation in CF is thought to be a consequence of mucus obstruction and chronic infection. However, in addition to CFTR’s well-established role in ion transport, there is growing evidence to suggest that CFTR interacts with, and modulates the function of, receptors involved in immune signaling [e.g., TNF-α, TLR4, and IFN-γ receptors^17, 42–45^]. This effect can be instrumental in regulating host immune responses.

We recently found that CF MΦs have blunted PI3K/AKT signaling in response to TLR activation^11^. A similar dysfunction was also observed in CF MΦs exposed to PA (Supplementary Fig. S5A). PI3K/AKT signaling fine-tunes the inflammatory response in MΦs by balancing the induction of signaling pathways that positively and negatively regulate inflammation^25^. For example, the PI3K/AKT pathway in MΦs has been implicated in mediating the stability of both IKK^46^ and Ik-B alpha^47^, which have opposite effects on NF-kB signaling. PI3K/AKT signaling promotes resolution of inflammation by: 1) negatively regulating TLR signal transduction^11, 27, 48^; 2) regulating levels of several micro-RNAs that determine the magnitude and duration of pro-inflammatory responses^11, 26,49^; and 3) controlling the polarization of macrophages^28^. PI3K/AKT signaling has also been implicated in *P. aeruginosa* host defense because it is required for sensing, and internalization, of bacteria into MΦs^40^. Finally, PI3K/AKT signaling controls cell polarity and chemotaxis by regulating the Cdc42/Rac effector kinase^50^, which was recently reported to be impaired in CF monocytes^12^. Thus, our finding of blunted PI3K/AKT activation could help to explain the phenotypic dysfunction associated with CF MΦs, including: hyper-inflammation, defective bacterial clearance, and defective migration.

In this study, we elucidated the mechanism by which loss of CFTR blunts PI3K/AKT signaling in activated murine and human MΦs. We identified Ezrin, an F-actin binding protein and component of the ERM protein family, as the link molecule between CFTR and TLR signaling in MΦs, which is required for efficient PI3K/AKT activation in response to LPS. Ezrin has been extensively studied in the context of epithelial cells. Its importance in regulating immune cell function has been demonstrated for T cells, where Ezrin modulates immunological synapse formation^51^, and for B cells, where it modulates humoral immunity by controlling B cell signaling and trafficking^52^. However, Ezrin’s role in MΦ immune responses has not yet been characterized.

Here, for the first time, we demonstrate that activation of TLR4 signaling in WT MΦs is accompanied by induction and activation (phosphorylation at the Thr 567 residue) of Ezrin and localization to filopodia. Using a genetic model, we showed that Ezrin is necessary for inducing PI3K/AKT phosphorylation in MΦs challenged with LPS and *P. aeruginosa* (Figs 2 and 3). Moreover, we identified the mechanisms by which Ezrin participates in host defense against *P. aeruginosa*. We observed impaired phagocytosis in MΦs lacking Ezrin (Fig. 3). This could be due to a primary effect, such as reduced engulfing capability that is caused by impaired plasma membrane function to surround bacteria. Alternatively, as we have shown (Fig. 3A), impaired phagocytosis could be a secondary consequence of the decreased Ezrin blunting PI3K/AKT signaling, which is required for sensing *P. aeruginosa* ^40^. Ezrin has also been implicated in regulating phago-lysosomal fusion^53^. This study used latex beads, but loss of Ezrin might also interfere with *P. aeruginosa* intracellular killing, which is a proposed characteristic of CF MΦs. Finally, with regards to PI3K/AKT signaling, Ezrin also promotes immune cell adhesion and migration, as shown in B cells^54, 55^. Thus, loss of Ezrin in CF MΦs might account for the recently described defect in migration^12^, for which a mechanism remains to be explored.

In conclusion, we have established the role of Ezrin as a key protein to orchestrate several important functions of MΦs including TLR4 signaling regulation, anti-inflammatory response, and host defense against *P. aeruginosa*. We also found that the presence of CFTR in MΦs is necessary for normal Ezrin induction/cellular localization in response to inflammatory triggers. We have described a major alteration of plasma membrane organization in activated CF MΦs, which disrupts a key signaling network, and thus promotes hyper-inflammation and weakens host defense. This alteration essentially recapitulates the clinically observed dysfunctions in CF lung disease. Therefore, an effective future long-term anti-inflammatory CF therapy will need to modulate monocyte/MΦ function. A deeper understanding of the molecular mechanism(s) behind CF MΦ immune dysfunction will assist to identify new therapeutic targets. Finally, these studies may be relevant to other chronic inflammatory lung diseases, particularly chronic obstructive pulmonary disease, which has been associated with systemic CFTR dysfunction^56^.

## Materials And Methods

### Constructs

To establish J774A.1-CTR and J774A.1-EZR cell lines, cells were transduced with four different retroviral vectors (RVs)-shRNA against Ezrin or control (http://www.origene.com/mouse_rna/TG502411.aspx). The pGFP-V-RS plasmid vector contains both 5- and 3- LTRs of Moloney murine leukemia virus (MMLV) flanking the puromycin marker and the U6-shRNA expression cassette. Retroviral preparations and virus titer assessment were performed using standard procedures^11^. For detailed information, please refer to the online supplemental experimental procedures.

### Chemicals and reagents

*Pseudomonas aeruginosa* (PA) LPS (Sigma-Aldrich) was prepared in PBS at 100X stock solution and used at a concentration of 10 µg/mL. Mg132 (Sigma-Aldrich) was dissolved in DMSO (stock solution 10 mM) and used at a final concentration of 10 µM.

### Isolation and culture of murine BMD and human peripheral-blood-derived macrophages

#### Murine macrophages

BM collection was performed as described previously^6^.

#### Human macrophages

Blood was obtained from healthy donors (HD) or from adult CF subjects (Supplementary Table 1) during routine clinic visits, with informed consent in accordance with relevant laws and institutional guidelines. These studies were approved by the Yale University Medical School Human Investigation Committee (HIC # 1307012431). Human MΦs were cultured as described previously^17^. For detailed information, please refer to the online supplemental experimental procedures.

### Cell transduction

J774A.1 cell lines were transduced at multiplicity of infection (MOI) virus:cells of 30:1. Briefly, 5 × 10^6^ cells were cultured in 6-well plates and exposed to each virus in 1 ml of serum-free medium. After spin infection, cells were incubated in serum-containing medium. Puromycin (10 µg/ml) was then used to select stably transduced J774A.1 cells, and Western blot (WB) and quantitative PCR (qPCR) for Ezrin were used to evaluate the silencing effect of the shRNA constructs 72 h post transfection (Supplementary Fig. S4).

### Mouse models and *in vivo* studies

All procedures were performed in compliance with relevant laws and institutional guidelines, and were approved by the Yale University Institutional Animal Care and Use Committee. Transgenic *Cftr* ^*−/−*^ (B6.129P2-KOCftr^tm1UNC^) mice were purchased from the Jackson Laboratory and bred in the Yale University Animal Facility. *Cftr* ^*−/−*^ mice are fully back-crossed to the C57BL/6 background. To improve survival and growth of *Cftr* ^*−/−*^ mice, which suffer strong susceptibility to intestinal obstruction, mice were fed on a liquid diet (Peptamen, Nestle, Deerfield, Illinois) starting at the time of weaning, as described in references^6, 10, 11, 57, 58^. Wild-type (WT) littermate mice used as controls were maintained on an identical diet to the *Cftr* ^*−/−*^ mice to eliminate nutritional status as a potential confounder. For the *in vivo* studies, mice were 4–5 months old (50% female, 50% male) and nebulized with *P. aeruginosa* (PA) LPS (12.5 mg), as previously described^6^. BM cells were isolated at 6–8 weeks of mouse age. Please refer to the online supplemental experimental procedures for details.

### RT-PCR and expression analysis

Cells were lysed in triazol (Qiagen), and total RNA was isolated from 1 × 10^6^ cells using QiagenRNAMini Kits^TM^ (Qiagen), following the manufacturer’s instructions. Real-time PCR analysis was performed with a Bio-Rad iCycler using TaqMan technology. Copy number was normalized by 18S and the relative expression to untreated cells was calculated by ∆∆Ct method. All TaqMan primers and probes were purchased from Applied Biosystems (Life Technology).

### Protein isolation and Western blot

Cells were washed three times with PBS, and total cell lysate was extracted using RIPA lysis buffer (Cell Signaling) containing 1mM phenylmethanesulfonyl fluoride (PMSF) and protease and phosphatase inhibitor cocktails (Roche Diagnostics). Protein concentration was determined by a Bradford assay and an equal amount of protein was separated by electrophoresis on 4–15% Mini PROTEAN Gels (Bio-Rad Laboratories, CA), transferred to nitrocellulose membrane (Bio-Rad Laboratories, CA), and probed with primary antibodies using standard procedures. pEzr/Ezr and pAKT/AKT ratios were calculated as follow: first, each WB band intensity was normalized for the corresponding β-Actin intensity. From the normalized values, we calculated the fold increase of treated to untreated samples. From these values, the pEzrin vs. total Ezrin and pAKT vs. total AKT ratios were calculated. For detailed information about antibodies used and WB analysis, please refer to the online supplemental experimental procedures.

### IF analysis

WT and CF MΦs were grown overnight on poly-L-lysine–coated 12-mm round coverslips at 70–80% confluence. The following day, cells were challenged with LPS. At the times indicated, cells were fixed for 15 min in 4% paraformaldehyde, permeabilized for 60 min in PBS/20%donkey serum/0.1% saponin, and stained, as indicated. Rabbit polyclonal anti-Ezrin (1:250, Abcam) antibody was used; directly conjugated fluorescent secondary antibody (ThermoFisher) was used at 1:300 dilutions, 1 h at room temperature. Pictures were taken with a confocal microscope (Leica TCS SP5 Spectral Confocal Microscope) using a 63X objective lens. Each image was subjected to Z-stack analysis. For each experiment, at least six different fields were acquired.

### EM morphology

The macrophages were fixed in Karnovsky Fixative for 1 h, washed in 0.1M Na cacodylate buffer pH 7.4 and post-fixed in 1% osmium tetroxide. They were in-block stained with uranyl acetate, then dehydrated and embedded in Embed 812 (Electron Microscopy Sciences). Ultrathin sections were cut on a Leica Ultracut UTC, then stained with uranyl acetate and Reynold’s lead. Images were taken on a Tecnai Biotwin Electron Microscope. For detailed information on the immunogold labeling, please refer to the online supplemental experimental procedures.

### Bacterial strain

Yellow fluorescent protein-labeled *P. aeruginosa* strain O1 (YFP-PAO1) was grown at 37 °C in Luria broth medium with 100 µg/ml gentamicin (Sigma-Aldrich). The expression of the YFP from the plasmid pMQ72 in PAO1 was induced by supplementing the medium with arabinose (0.2%). For the CFU assay, the original PAO1 strain lacking gentamicin resistance was used. PAO1 strains were grown as previously described^59^. Briefly, bacteria were grown to late log phase, washed, re-suspended in DMEM + 10%FBS and added to J774A.1 cell lines at the indicated multiplicities of infection.

### Phagocytosis assay

Overnight culture of YFP-PAO1 was re-suspended in antibiotic-free DMEM medium containing 10% heat-inactivated FBS. J774A.1-CTR or J774A.1-EZR cell lines were seeded in 3.3 cm dishes (1 × 10^6^) and incubated with YFP-PAO1 at an MOI bacteria:cells of 100:1. After 30 min, phagocytosis was stopped by transferring cells to ice. Cells were washed in PBS, incubated with Fc block (BD Pharmingen) for 5 min on ice, and labeled with rat anti-mouse CD45-APC-Cy7 (Clone:30-F11, BD Pharmingen) for 30 min. All samples were acquired on an ImageStream®X Mark II using 488 and 642 nm laser. A total of 10000 events were collected for each sample. The IDEAS software (Amnis Corporation, Seattle Wa) was used to assess the phagocytosis index. For details of the ImageStream gating strategy (Fig. 3D), please refer to the online supplemental experimental procedures. Phagocytosis was further confirmed by colony-forming unit (CFU) assay (PAO1 at an MOI of 20:1), performed using standard procedure described in the online supplemental experimental procedures.

### Statistical Analysis

Statistical analyses were conducted using two-tailed two-sample t-tests or two-sample unequal variance t-tests. All experiments were performed in triplicate, unless otherwise indicated. Data are expressed as mean ± standard deviation. A *P* value < 0.05 was considered statistically significant. Prism 7.0 (GraphPad) was used for all statistical analyses.

## Electronic supplementary material

Ezrin links CFTR to TLR4 signaling to orchestrate anti-bacterial immune response in macrophages
