# Supplementary material for: Ezrin links CFTR to TLR4 signaling to orchestrate anti-bacterial immune response in macrophages
Source: Sci Rep. 2017 Sep 7;7:10882. doi: 10.1038/s41598-017-11012-7 (PMC5589856; doi:10.1038/s41598-017-11012-7)

Supplementary Information

Title:

Ezrin links CFTR to TLR4 signaling to orchestrate antibacterial immune response in macrophages

Authors:

Caterina Di Pietro[1], Ping-xia Zhang[1], Timothy K O'Rourke Jr.[1,6], Thomas S Murray[2,6], Lin Wang[2], Clemente J Britto[3], Jonathan L Koff [3], Diane S Krause[2,4], Marie E Egan[1,5] and Emanuela M Bruscia[1] *

Departments of Pediatrics[1], Laboratory Medicine[2], Internal Medicine[3], Pathology[4] and Cellular and Molecular Physiology[5], Yale University School of Medicine, New Haven CT, USA; Quinnipiac University Frank H. Netter MD School of Medicine [6], Hamden, CT, USA

***Corresponding Author:**

Emanuela M. Bruscia, Ph.D.
Associate Professor
Department of Pediatrics
Yale University School of Medicine
330 Cedar Street, Room 503
New Haven, Connecticut 06520
Phone: (203) 737-5556
FAX: (203) 785-6337
e-mail: emanuela.bruscia@yale.edu

Supplementary Experimental Procedures

Retroviral Preparation and Virus Titer assessment:

Packing cells 293T ($5x10^6$) were transfected with plasmid mixture containing 10 µg of retroviral construct, 10 µg of gag/pol, and 10 µg of VSVG plasmid constructs using FuGENE HD transfection reagent (Promega) following the manufacturer's instructions. Retroviruses were harvested in viral collection medium (DMEM with 10% heat-inactivated fetal bovine serum +1% penicillin-streptomycin-glutamine+20mM HEPES) for 3 days after transfection, twice a day. The viral collection medium was spun down at 1,500 rpm for 5 min to remove 293T cells and concentrated with Amicon Ultra centrifugal filter-10K (Millipore), at 4,000 rpm for 30 min at 4 °C. Then, viral vectors were aliquoted and stored at -80 °C until use. Retrovirus titer was assessed using 293T cells. $1X10^6$ cells were plated in 1.25 ml medium (DMEM supplemented with 10% heat-inactivated FBS; antibiotics and L-glutamine) together with 8 µg/ml of polybrene (American Bioanalytical) and with RVs at different dilutions (0, 5 µl, 15 µl, and 30 µl)

Isolation and culture of murine bone-marrow-derived (BM-derived) and human peripheral-blood-derived macrophages

Murine macrophages: BM cells were flushed with DMEM medium from the medullary cavities of the tibias and femurs using 25G needle in sterile conditions. The BM suspension was filtered through a 70 µm cell strainer, then mononuclear cells were enriched by the Ficoll-Paque method (Histopaque 1077 Sigma H8889). After overnight culture, the non-adherent cells were differentiated for 7 days in 20 ng/ml recombinant M-CSF (ConnStem Inc., CT, USA). After 7 days, cells were detached and characterized by flow cytometry (F4-80+/MAC-1+ population). The day before experiments, cells were plated according to experimental design.

Human macrophages: Human mononuclear cells were isolated from whole blood by the Ficoll-Paque method (Histopaque 1077 Sigma H8889), and seeded at $5x10^6$ cells/well in 6 well-plates in RPMI supplemented with 10% FBS and 40 ng/ml recombinant human M-CSF (ConnStem Inc., CT, USA). Cells were fed every other day and split 1:1 every 4 days. After 1-2 weeks, cells were characterized by flow cytometry (CD14+/CD45+) and the morphology analyzed on cytospin. Before LPS treatment, cells were washed extensively with PBS. About $1x10^6$ cells/well (6 well-plates) were treated with LPS as indicated.

Macrophage J774A.1 cell lines: were cultured in DMEM supplemented with 10% FBS and 1% penicillin-streptomycin-glutamine at 37 °C in the presence of 5% CO_2. The media of J774A.1 cells stably infected with retroviral vectors expressing sh-RNA against Ezrin (or scramble sh-RNA) were maintained in 10 µg/ml of puromycin (Gibco).

Mouse models and in vivo studies

WT or CF mice received *Pseudomonas aeruginosa* (PA) LPS (Sigma L8643) over 3 days (a dose a day). LPS (12.5 mg) was administered with a nebulizer (Pulmo-Aide Compressor, Natallergy). Five ml of solution were

nebulized over 15 min. Mice were sacrificed 24 h post LPS nebulization. After cardiac perfusion with PBS supplemented with heparin, the right lobes were snap frozen in liquid nitrogen and used for protein isolation.

Western Blot Antibodies

The following antibodies were used: rabbit anti-HO-1 (1:2000, Abcam), rabbit polyclonal anti-AKT and anti-pAKT (1:1000, Cell Signaling Technology), rabbit polyclonal anti-Ezrin (1:1000, Abcam) and pEzrin (Thr567), (1:1500, SAB), mouse monoclonal GADPH (1:1000, Novus), and rabbit-HRP anti-actin (1:5000, Santa Cruz). For detection, horseradish peroxidase was conjugated to IgG secondary antibodies (1:2000, Santa Cruz), followed by visualization using enhanced chemiluminescence (ECL). The chemiluminescence imaging system ChemiDoc (Biorad) and the Image lab software (Biorad) were used for image acquisition and for signal quantification. Protein relative expression is normalized to β-Actin. Images have been cropped for presentation.

Immunogold Labeling

MΦs were grown on Thermanox Coverslips (Nalge Nuc International). They were fixed overnight in 4% paraformaldehyde in 0.1 M phosphate buffer. They were washed in PBS and antigen retrieved in citrate buffer at 40% power for 15 s intervals for 1 min, then cooled in the citrate for 20 min. The aldehydes were quenched in 50 mM glycine for 20 min. After blocking and permeabilizing in 2% BSA, 0.1% cold water fish gelatin and 0.1% Tween 20 in PBS for 1 h, they were incubated in Ezrin (Abcam) primary antibody diluted 1/25 in PBS 0.1% BSA, 10% goat serum, and 0.05% Tween 20, and kept overnight at 4 °C. After washing in PBS, they were incubated in secondary 10 nm gold conjugate at 1/50 dilution. MΦs were then post-fixed in 2.5% glutaraldehyde in 0.1M phosphate buffer for 30 min. To enhance the contrast, they were treated in 1% osmium tetroxide with potassium ferrocyanide. They were rinsed in water and in-block-stained with uranyl acetate. Next, they were dehydrated and embedded in Embed 812. Ultrathin sections were cut and stained in uranyl acetate and Reynold’s lead stain. The images were taken on the Tecnai biotwin Electron Microscope.

ImageStream and CFU assay

Overnight cultures of YFP-PAO1 or PAO1 were washed and re-suspended in antibiotic-free DMEM +10% FBS, and bacterial concentration was determined. J774A.1-CTR or J774A.1-EZR cell lines were seeded ($1x10^6$) and allowed to adhere overnight prior to infection with the bacteria at an MOI bacteria:cells of 100:1 (Imagestream) or 20:1(CFU) for 30min.

ImageStream: To assess phagocytosis, cells were detached, stained for CD45 and fixed with PFA (2%). The gating strategy to quantify phagocytosis with the ImageStream is outlined in supplementary **Fig. 3D**: first, objects with high gradient RMS (root mean square for image sharpness) value were gated to obtain cells in best focus (i). To limit the analysis to single cells, debris and multi-cellular events were eliminated by using the IDEAS features *area* and *aspect ratio* of the bright field image (M01) (ii). Thereafter, a gate was set on single cells expressing CD45 marker (iii). To discriminate between internalized from cell surface-bound bacteria, we created an internalization mask: $CD45^+$ cells were plotted based on intensity of YFP (Channel 2, x-axis) versus Max

Pixel of YFP (Channel 2, y-axis), which allowed to draw a region around cells associated with bacteria (iv). A mask representing the whole cell was created based on plasma membrane CD45 staining (Channel 12) first, and then eroded by 5 pixels (v). Bacteria are considered internalized when fluorescence signal is within the mask. The internalization mask was then applied to all YFP^+ cells to calculate the internalization score (vi). Cells with internalized bacteria were selected by choosing the cell population with an internalization score equal to or greater than 0, an arbitrary value selected by viewing the images in selected bin mode. Cells receiving a score less than 0 were considered to be surface bound. The number of cells within the internalization gate, automatically generated by the software, was then used to calculate the phagocytosis index by dividing this number by the total $CD45^+$ cells. Finally, cells within the internalization gate were further characterized based on the number of bacteria ingested by using the Spot Count feature. All samples were acquired on an ImageStream®X Mark II using 488 and 642 nm laser. A total of 10000 events were collected for each sample. The IDEAS software (Amnis Corporation, Seattle Wa) was used to assess the phagocytosis index.

CFU assay: After 30 min of incubation at 37°C, gentamicin was added to the medium at 100 μg/ml for 40 min to kill extracellular bacteria. Next, cells were washed with PBS for three times and re-suspended in PBS containing 0.1% Triton X-100 to lyse cells and release intracellular bacteria. Samples were plated on LB-agar, and colony-forming units were counted after overnight incubation at 37°C.

Supplementary Figures and Tables

Supplementary Figure S1, related to Fig. 1. (**A**) qPCR for Ezrin in WT and CF murine BMD MΦs untreated or challenged with LPS at the time indicated. Ezrin expression is normalized to S18. (**B**) Bar graph representing the pEzrin/Ezrin protein expression ratio in murine WT (white bars) and CF (red bars) BMD MΦs, untreated or treated with LPS for the times indicated. (**C**) Bar graph representing the pEzrin/Ezrin protein expression ratio in lung lysates from WT (white bars) and CF (red bars) mice 24 h post LPS nebulization. Unless otherwise indicated, for each experiment, graphs represent the mean value of three biological repeats. Statistical analyses were conducted using one-sided two-sample *t*-tests. Error bars indicate standard deviation. Symbol *indicates a statistically significant difference between the experimental group and control group (*P* <0.05).

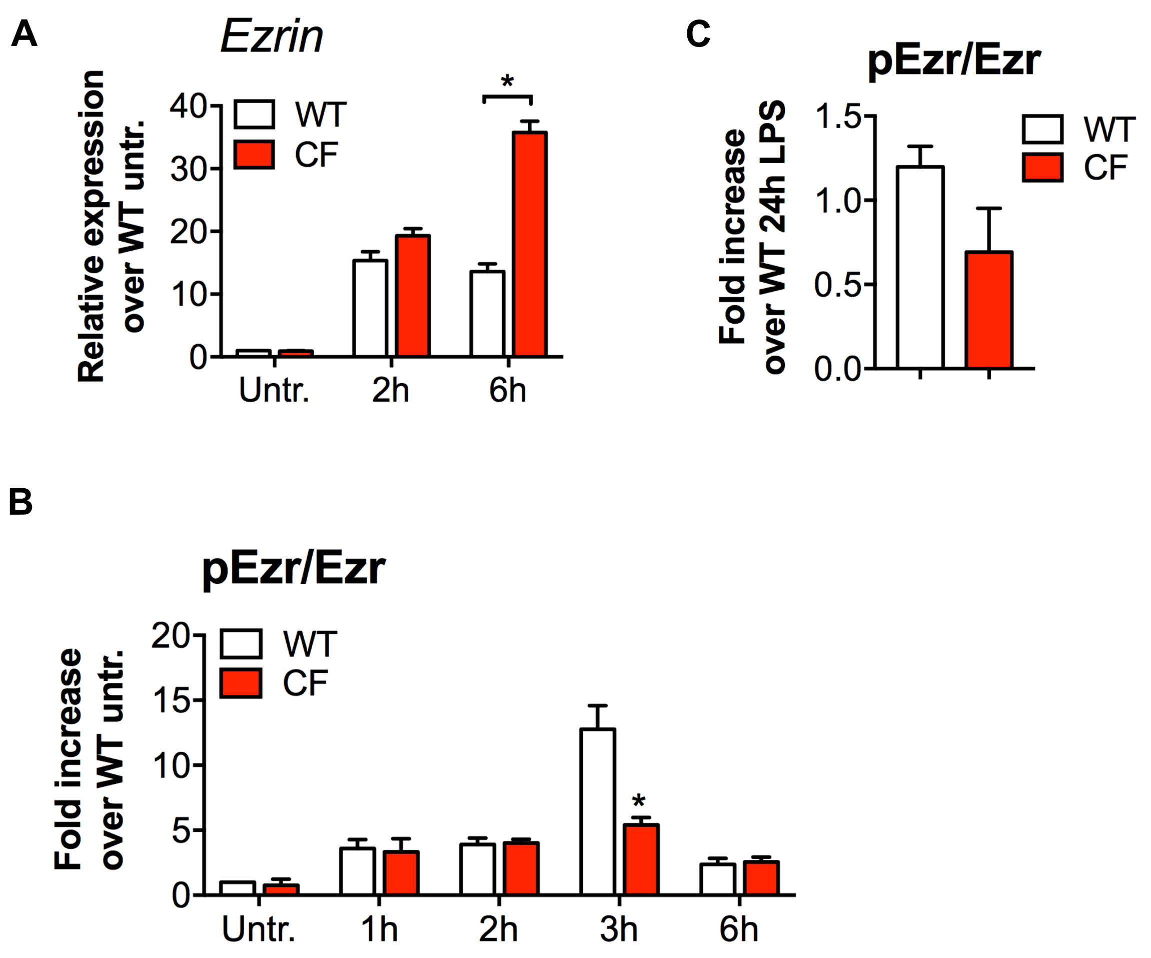

Supplementary Figure S2, related to Fig. 1. Representative TEM images for murine WT and CF BMD MΦs treated with LPS (6 h). The images are representative of three experimental biological repeats.

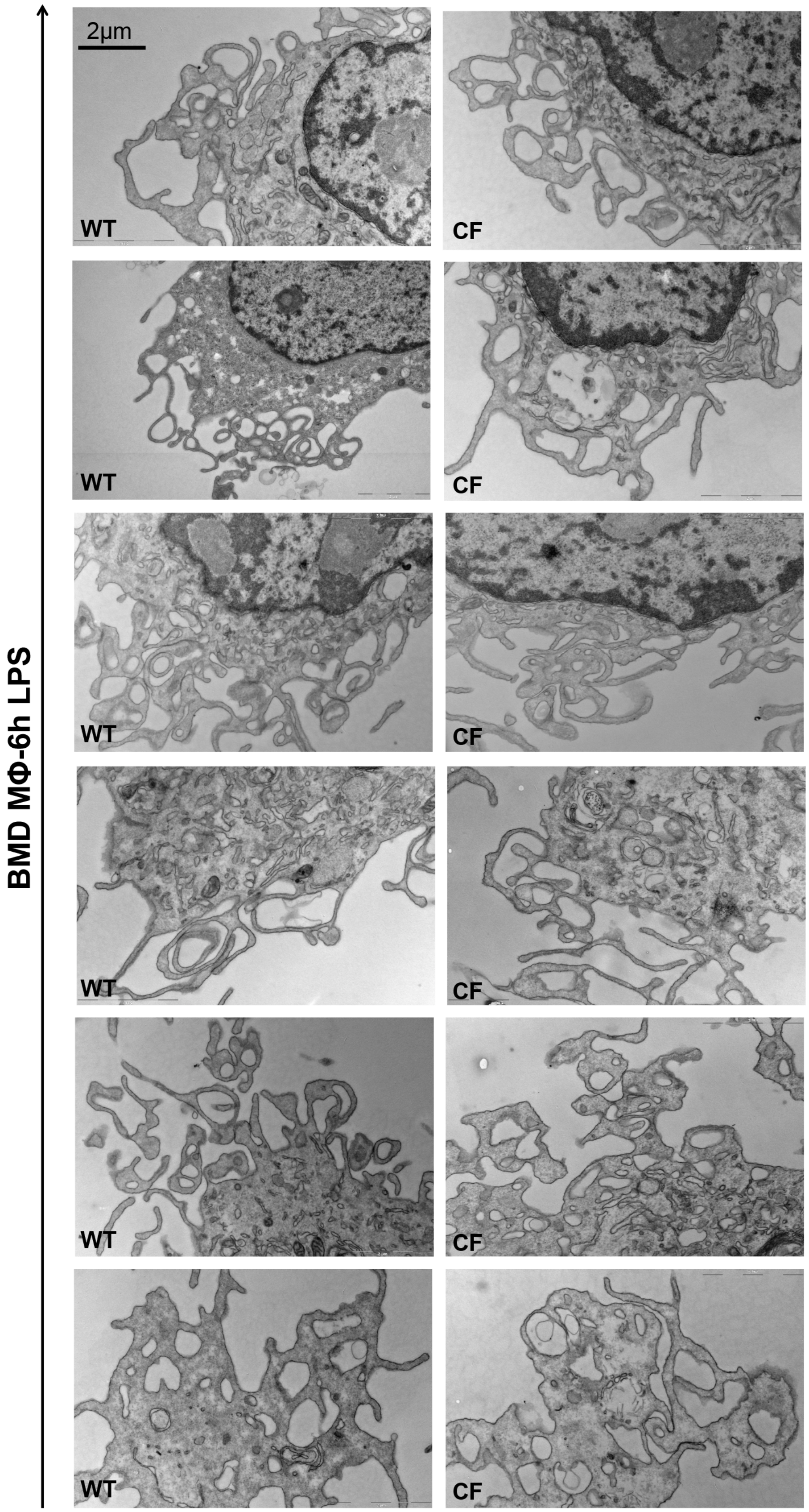

Supplementary Figure S3, related to Fig. 1. Representative Ezrin immunogold images for murine WT and CF BMD MΦs treated with LPS (6 h). The images are representative of three experimental biological repeats. Red arrows indicate gold staining.

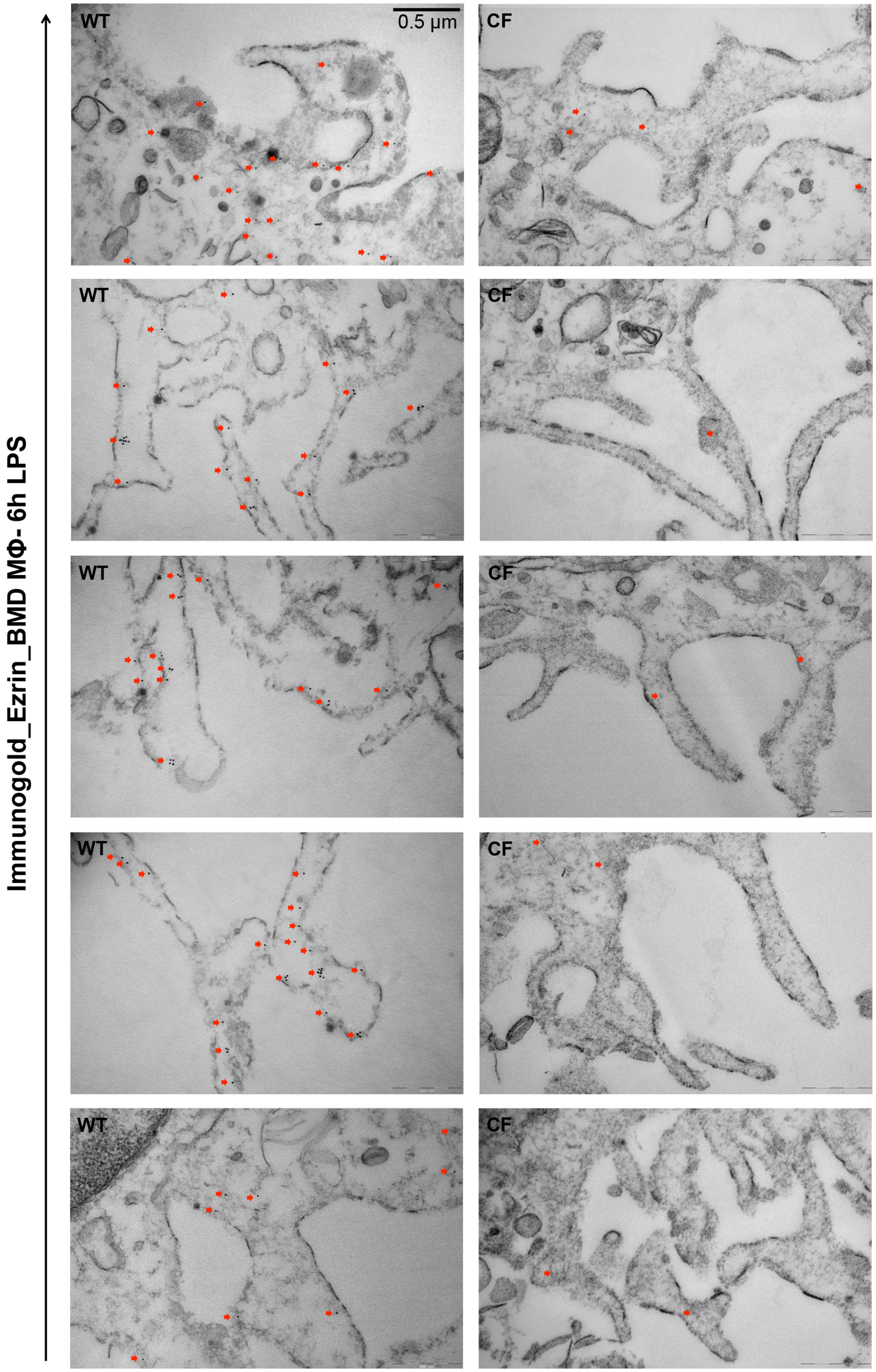

Supplementary Figure S4, related to Fig. 2. **(A)** WB and densitometric analysis for total Ezrin in J774A.1 cells stably transduced with retroviral vector (RV) expressing shRNA control (shRNA-CTR) or four different RV-shRNAs against Ezrin (Sh-673, Sh-674, Sh-675, Sh-676). (**B**) qPCR and (**C**) WB and densitometric analysis for Ezrin in murine J774A.1 expressing the shRNA control (shRNA-CTR, white bars) or the shRNA-676 (purple bars) untreated or treated with LPS at the time indicated. (**D**) qPCR for Ezrin in murine J774A.1 expressing the shRNA control (shRNA-CTR, white bars), shRNA-674 (green bar) or shRNA-676 (purple bars) treated 4h with LPS. (**E)** Densitometric analysis for pAKT and total AKT in murine J774A.1 cell lines transduced with Sh-674 or the shRNA-CTR, untreated or treated with LPS. Bar graph represents the pAKT/AKT ratio. **(F)** qPCR for IL-6 (left) and TNF-α (right) in shRNA-CTR or shRNA-674 J774A.1 cell lines, untreated or treated with LPS. For WB protein fold increase is normalized to β-Actin. For qPCR Ezrin, IL-6 and TNF-α expression is normalized to S18. Graphs represent the mean value of three biological repeats. Statistical analyses were conducted using one-sided two-sample *t*-tests. Error bars indicate standard deviation. Symbol *indicates a statistically significant difference between the experimental group and control group (*P* <0.05).

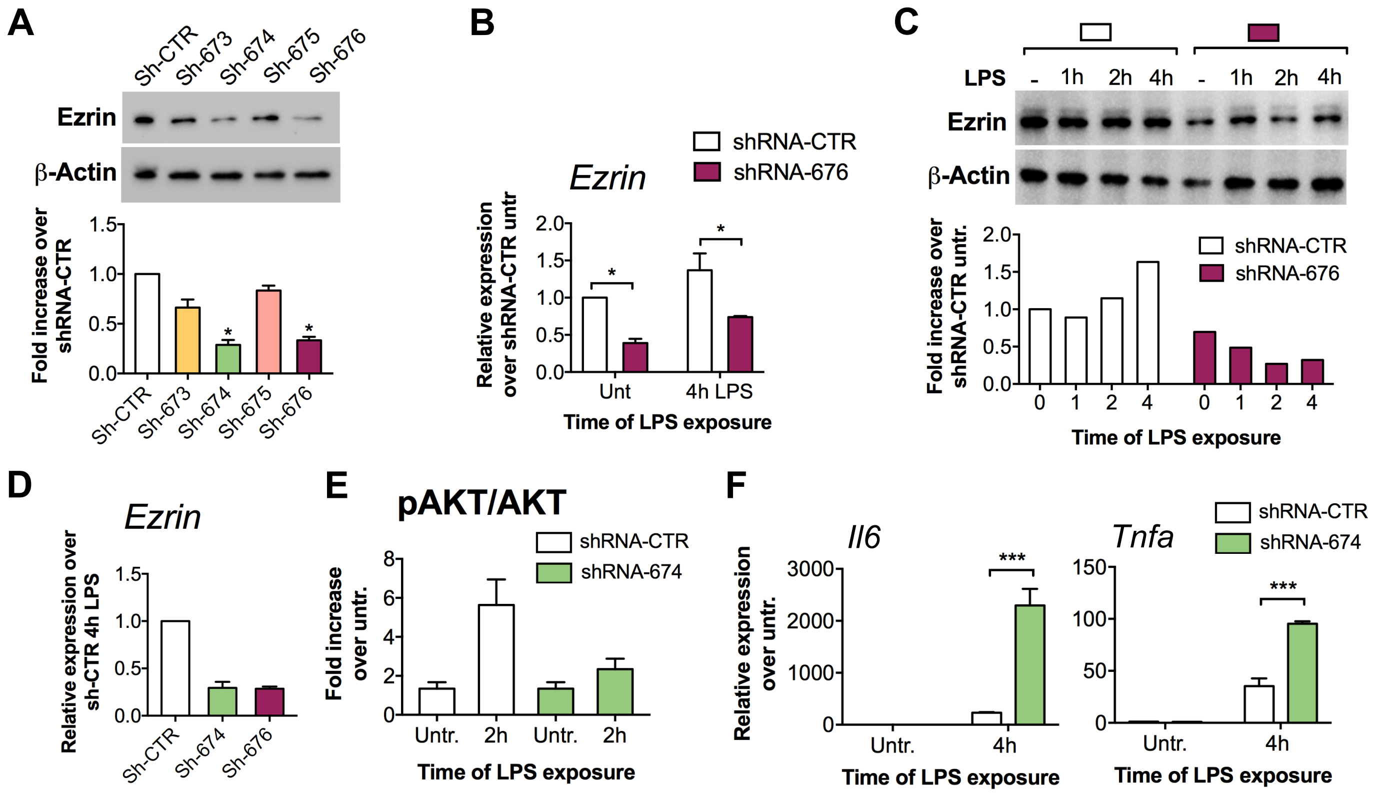

Supplementary Figure S5, related to Fig. 3. (**A**) WB and densitometric analysis for pAKT and total AKT in murine WT (white bars) and CF (red bars) BMD MΦs, untreated or treated with PAO1 (MOI:20) for 30 min. **(B)** WB and densitometric analysis for total Ezrin in J774A.1 cells stably transduced with RV- shRNA-CTR (shRNA-CTR, white bars) or RV-shRNAs-676 (shRNA-EZR, purple bars) untreated or treated with PAO1 (MOI:20) for 1h. **(C)** Representative ImageStream images of J774A.1 shRNA- shRNA-EZR used for phagocytosis analysis (30 min after PAO1 exposure). **(D)** Cells in the "internalized" gate (YFP-PAO1) were further characterized based on the number of spots (bacteria). Representative plots of cells with 1 or more ingested PAO1 (upper panel) and the corresponding bar graphs (bottom panel) are shown. The images are representative of three biological repeats. Error bars indicate standard deviation. Symbol * indicates a statistically significant difference between the experimental group and control group (*P* <0.05).

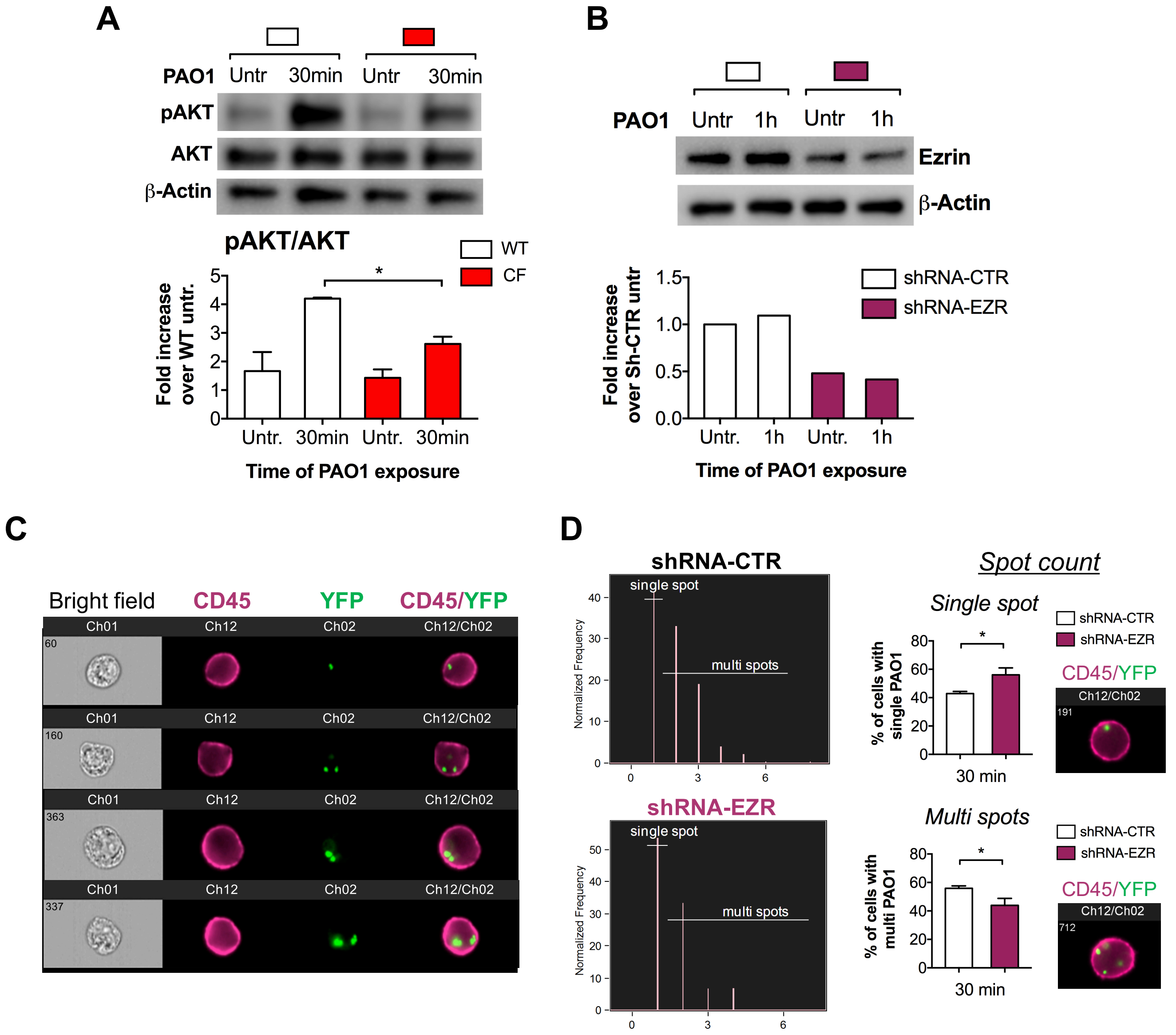

Supplementary Figure S6, related to Fig. 4. WB and densitometric analysis for pEzrin, Ezrin and pEzr/Ezr in PBD MΦs from each HD and CF experimental pair untreated or treated with LPS for the times indicated.

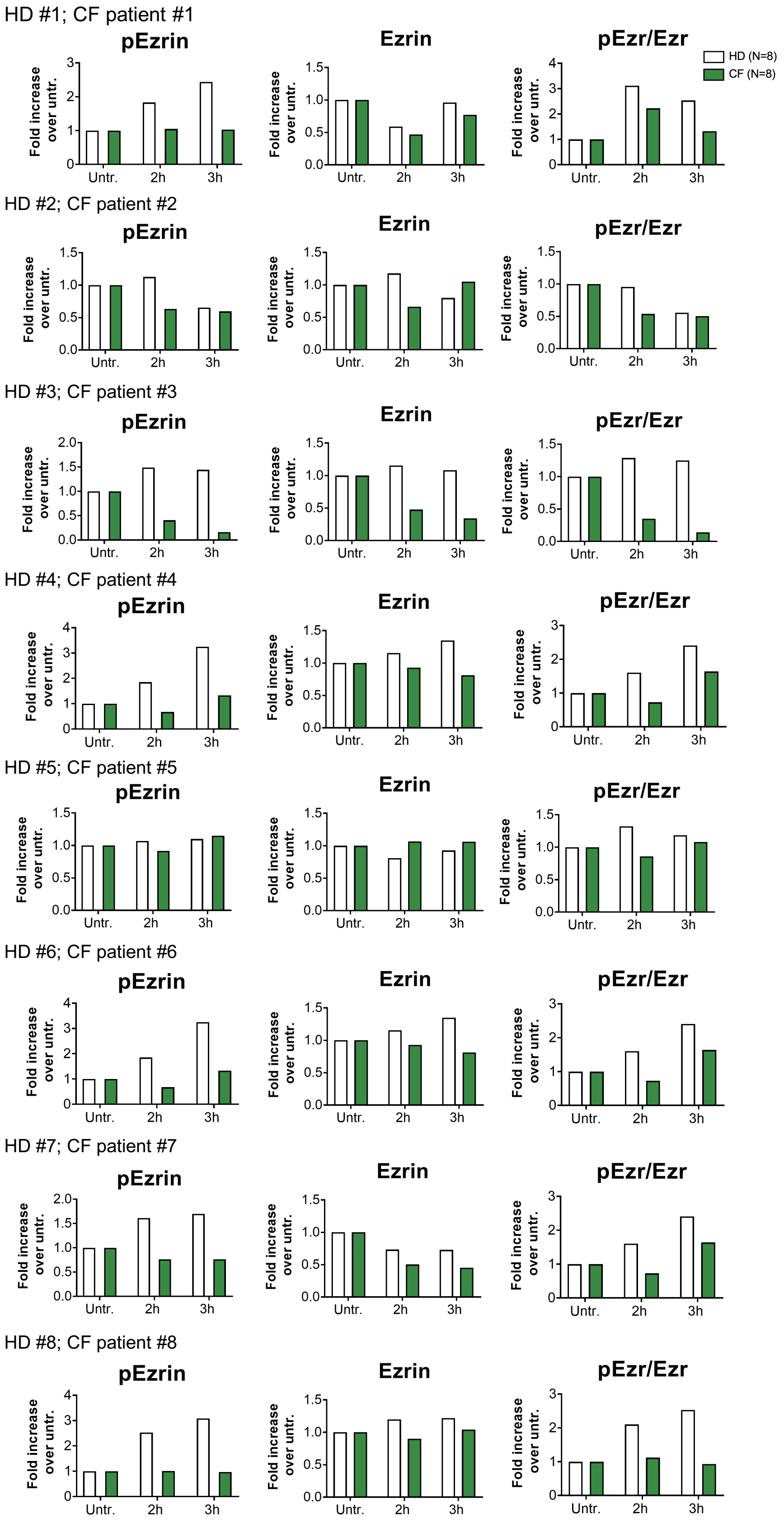

Supplementary Figure S7, related to Fig. 4. Densitometric analysis for pAKT, AKT and pAKT/AKT in PBD MΦs from HD and CF experimental pair untreated or treated with LPS for the times indicated.

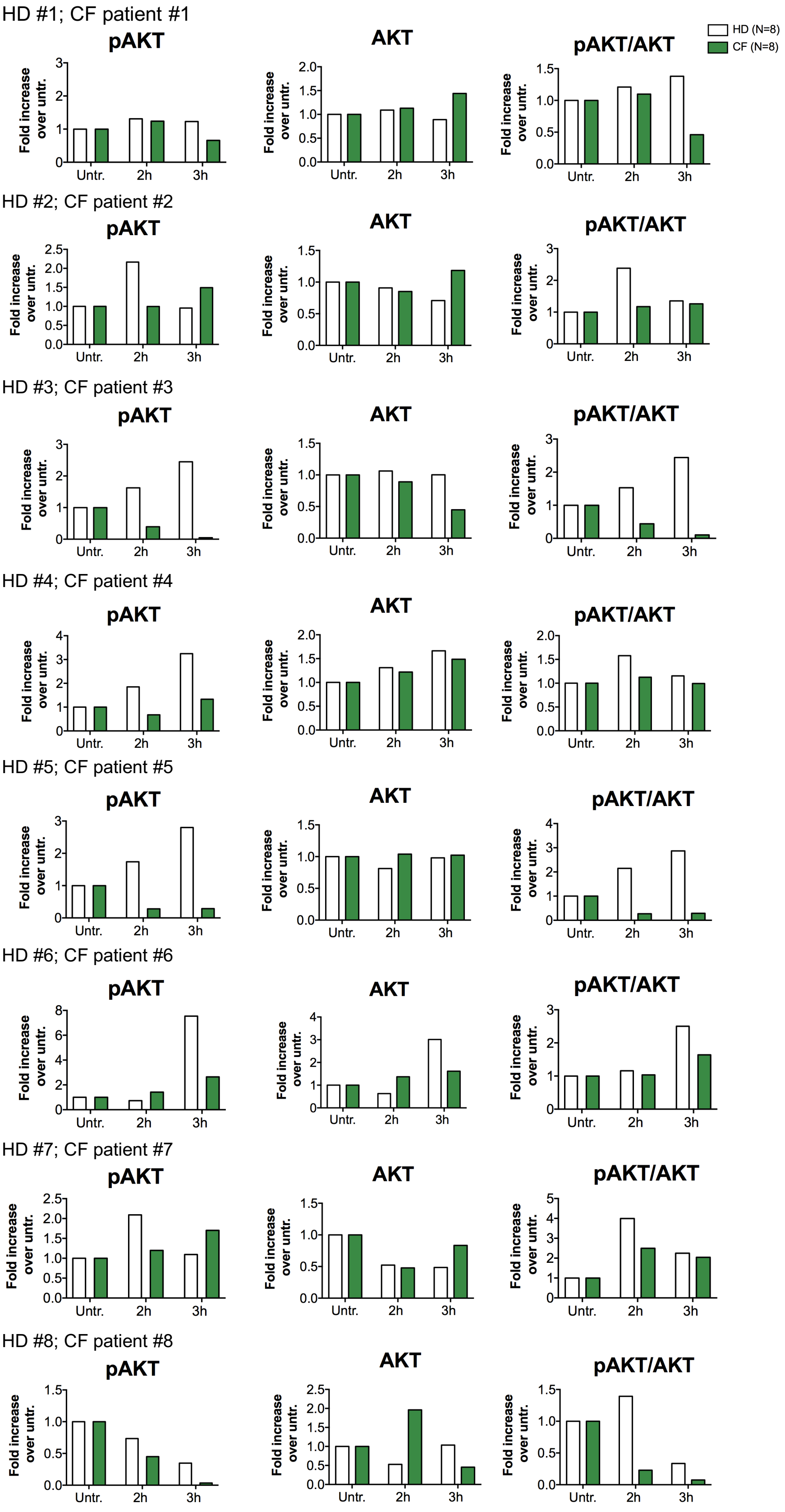

Table S1, related to Fig. 4. Genetics, baseline demographic, and clinical observations for CF subjects.

	Age	***Gender***	***Genotype***	***FEV1***	***PI***	***Below goal BMI***	***CFRD***
1	**25**	**F**	**f508del/f508del**	**0.72 (19%)**	**Yes**	**Yes**	**No**
2	**22**	**F**	**f508del/f508del**	**2.60 (78%)**	**Yes**	**Yes**	**Yes**
3	**29**	**M**	**f508del/ G542X**	**2.34 (59%)**	**Yes**	**No**	**No**
4	**32**	**M**	**f508del/f508del***	**3.45 (80%)**	**Yes**	**No**	**Yes**
5	**35**	**M**	**f508del/W1282X**	**1.12 (27%)**	**Yes**	**No**	**No**
6	**35**	**M**	**f508del/W1282X**	**1.18 (28%)**	**Yes**	**No**	**No**
7	**41**	**M**	**f508del/CF deletion E.23**	**1.29 (32%)**	**Yes**	**Yes**	**Yes**
8	**28**	**M**	**f508del/f508del**	**1.35 (32%)**	**Yes**	**Yes**	**Yes**

PI = pancreatic insufficient

Goal BMI = >22 women; >23 men

*** Lumacaftor**

SUPPLEMENTARY FIGURE 8A
(related to Figure 1B)

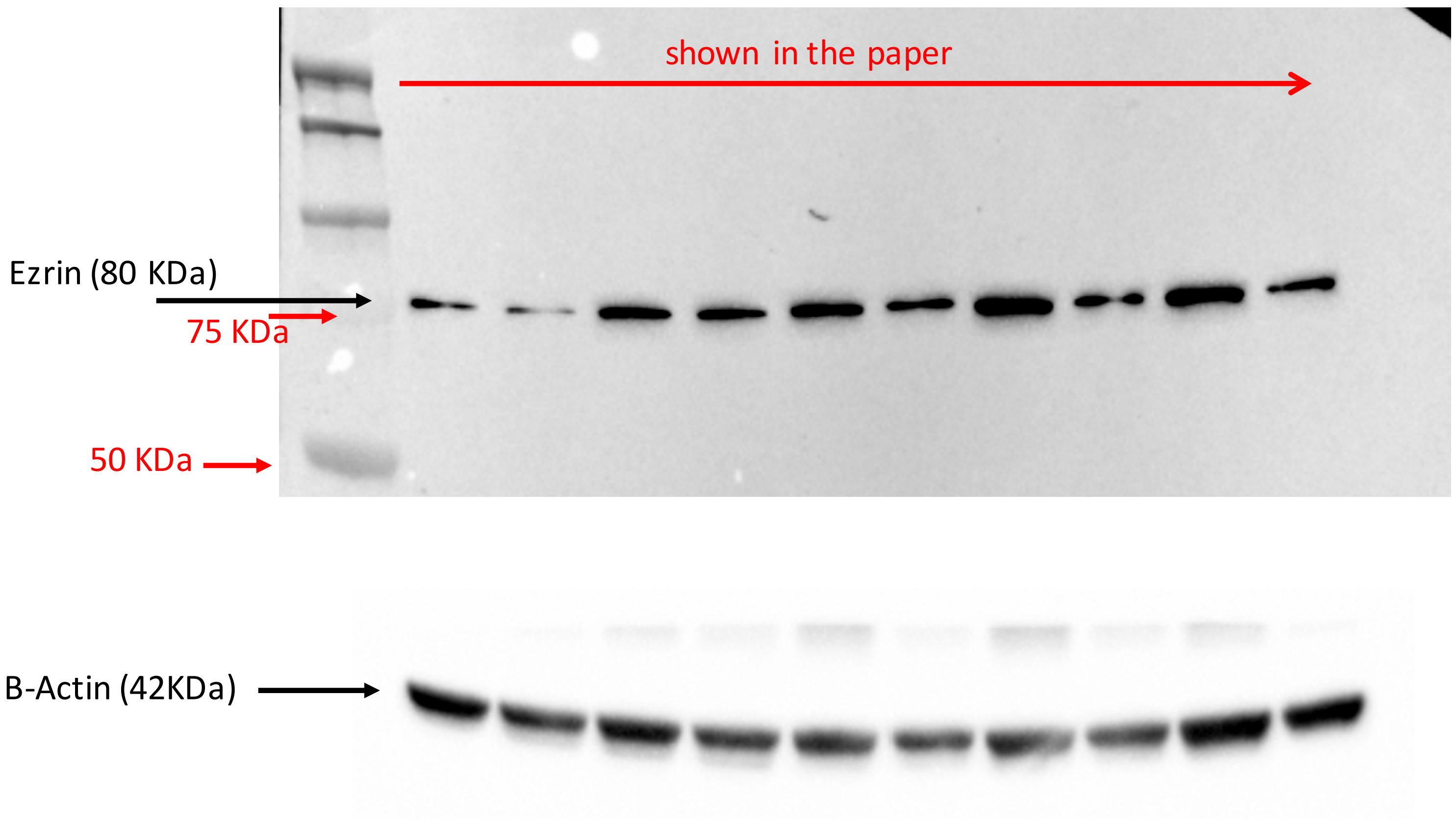

SUPPLEMENTARY FIGURE 8B
(related to Figure 1C)

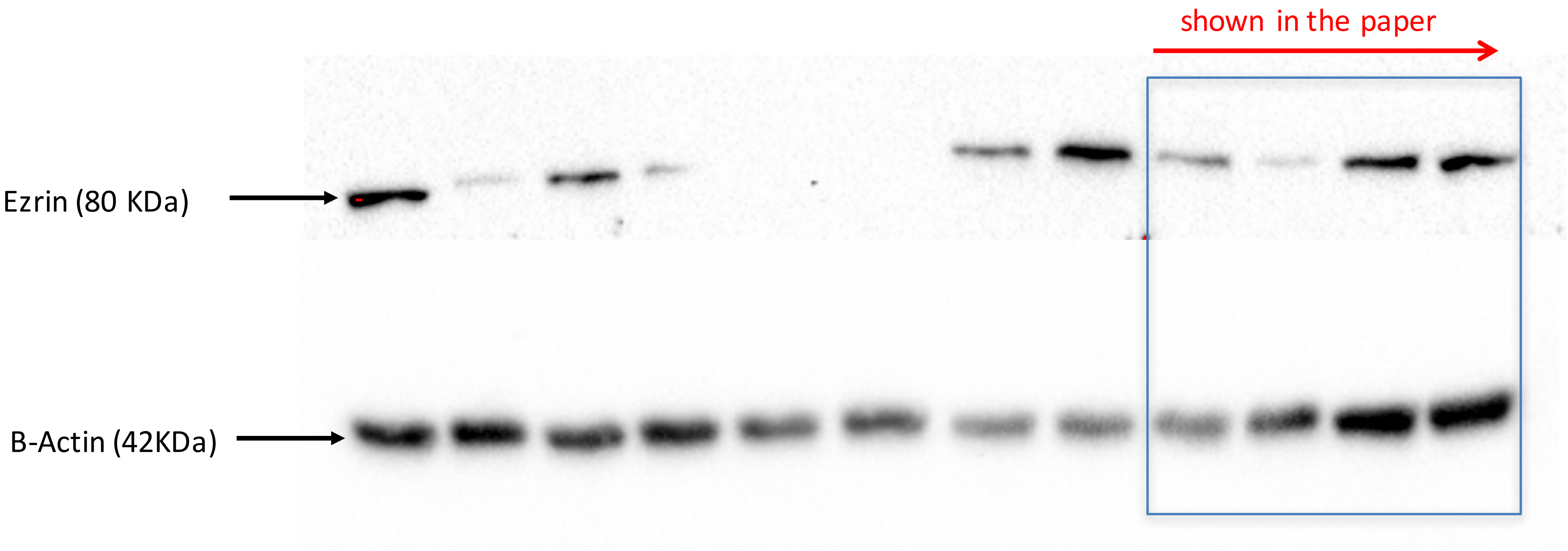

SUPPLEMENTARY FIGURE 8C
(related to Figure 1D)

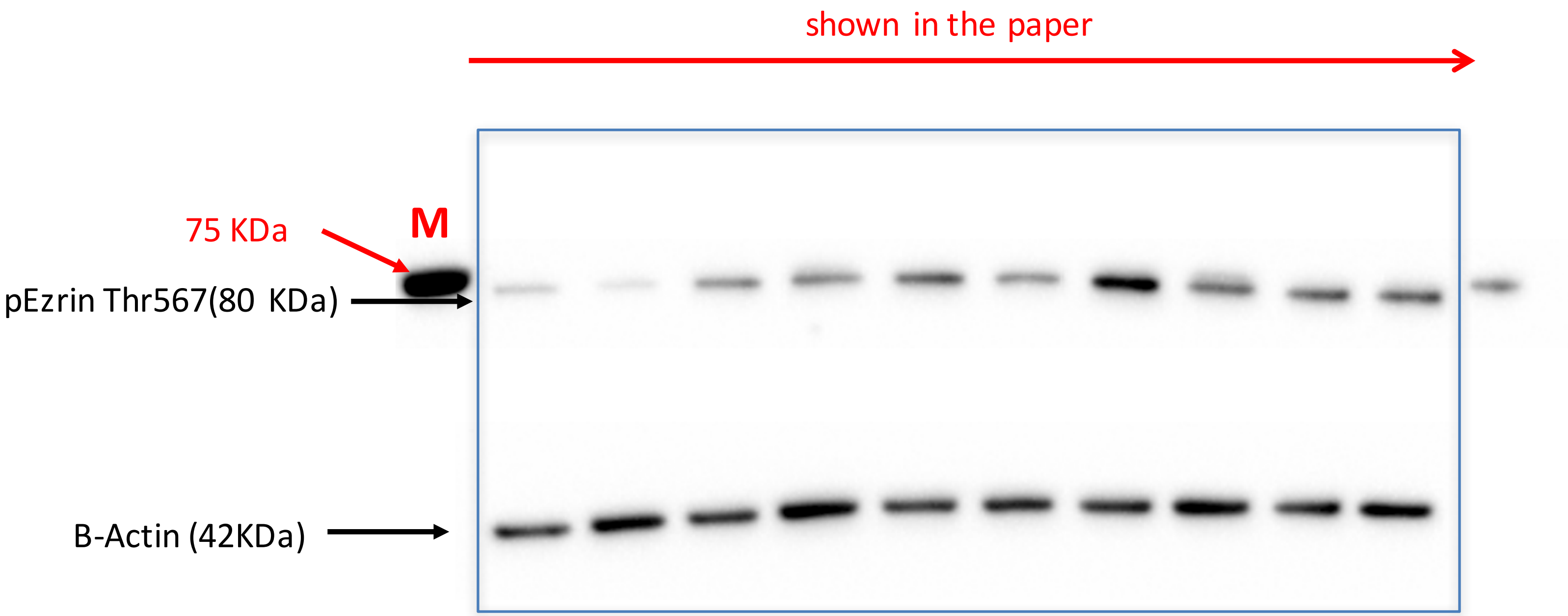

SUPPLEMENTARY FIGURE 8D
(related to Figure 1G)

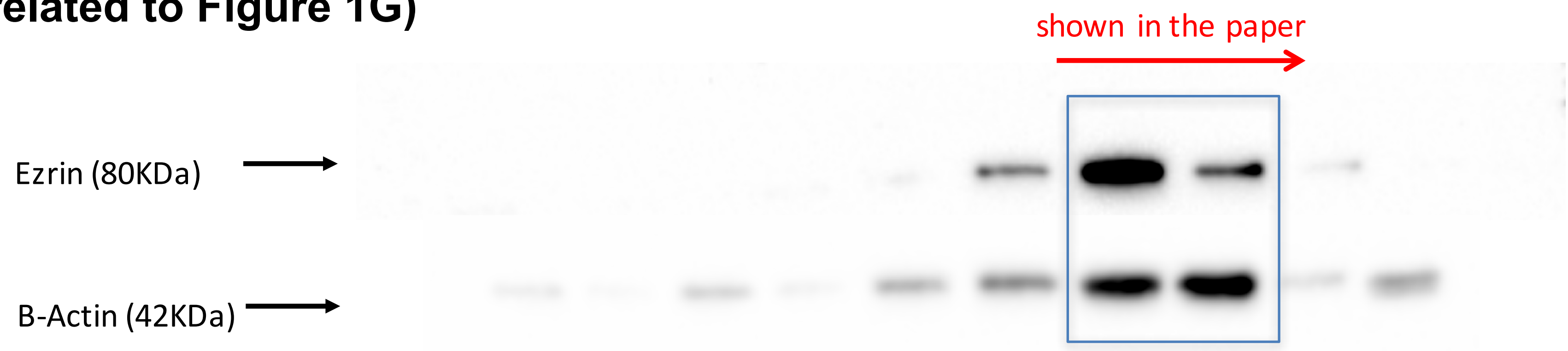

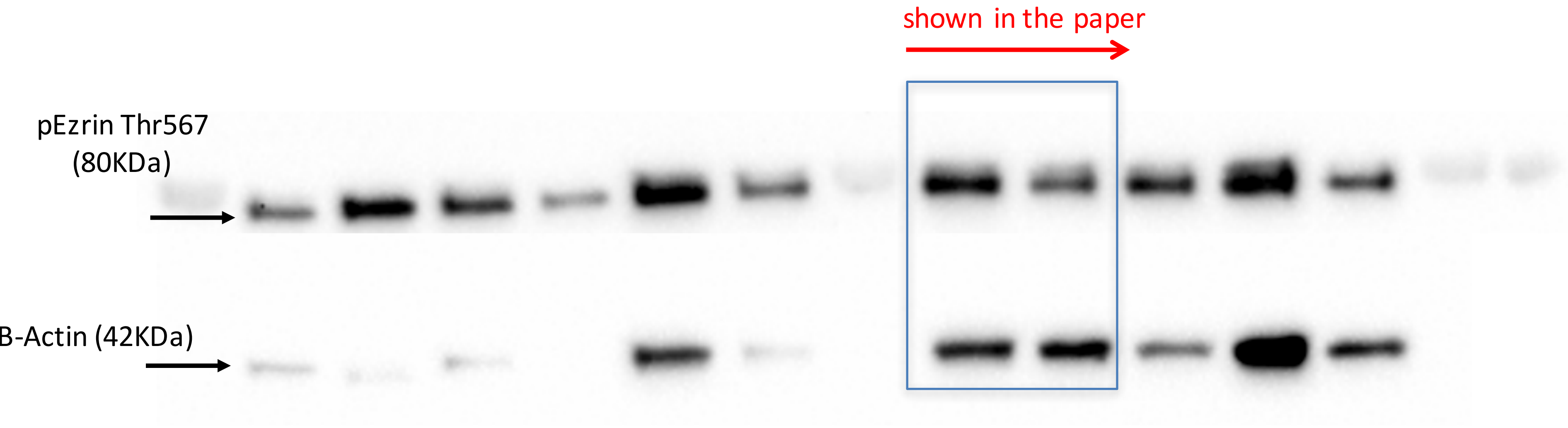

SUPPLEMENTARY FIGURE 8E
(related to Figure 2A)

GEL 1

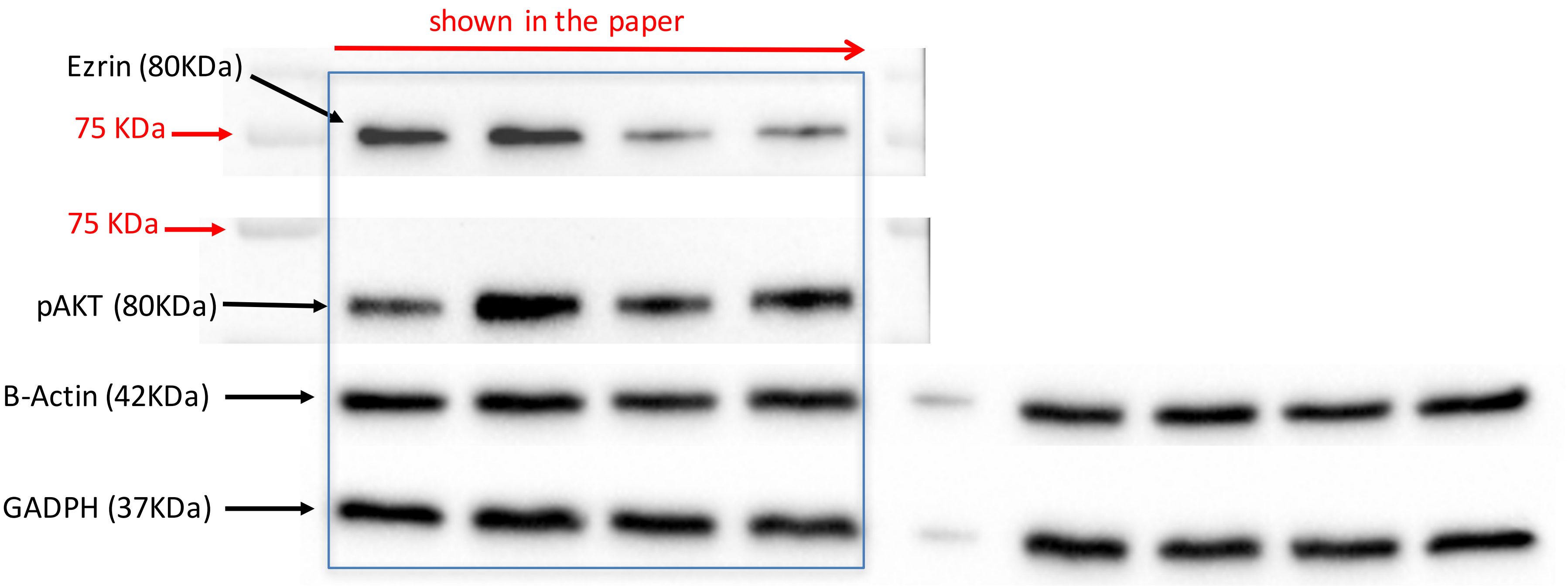

GEL 2

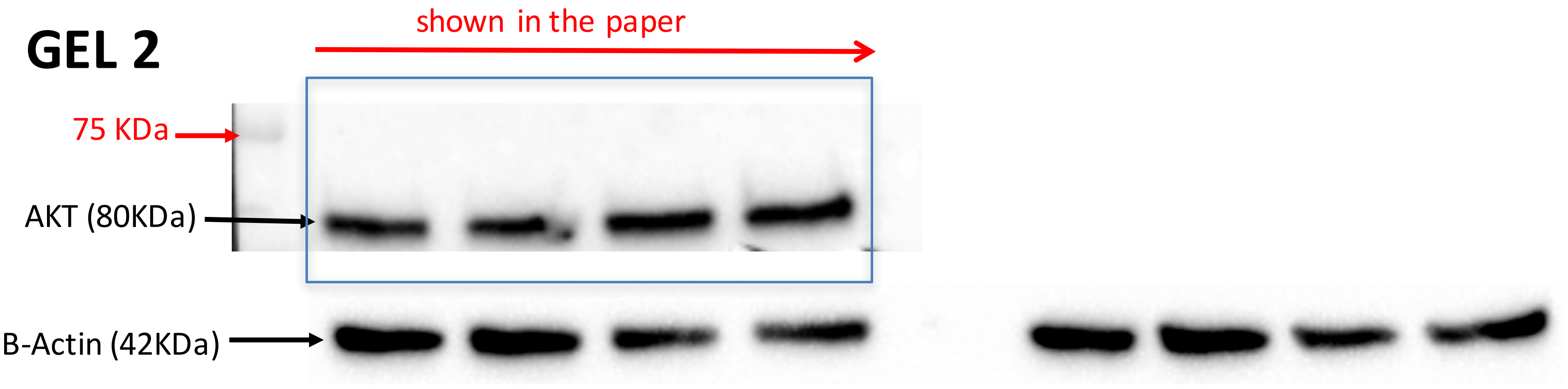

Membranes were cut before incubation with primary antibodies

SUPPLEMENTARY FIGURE 8F
(related to Figure 2C)

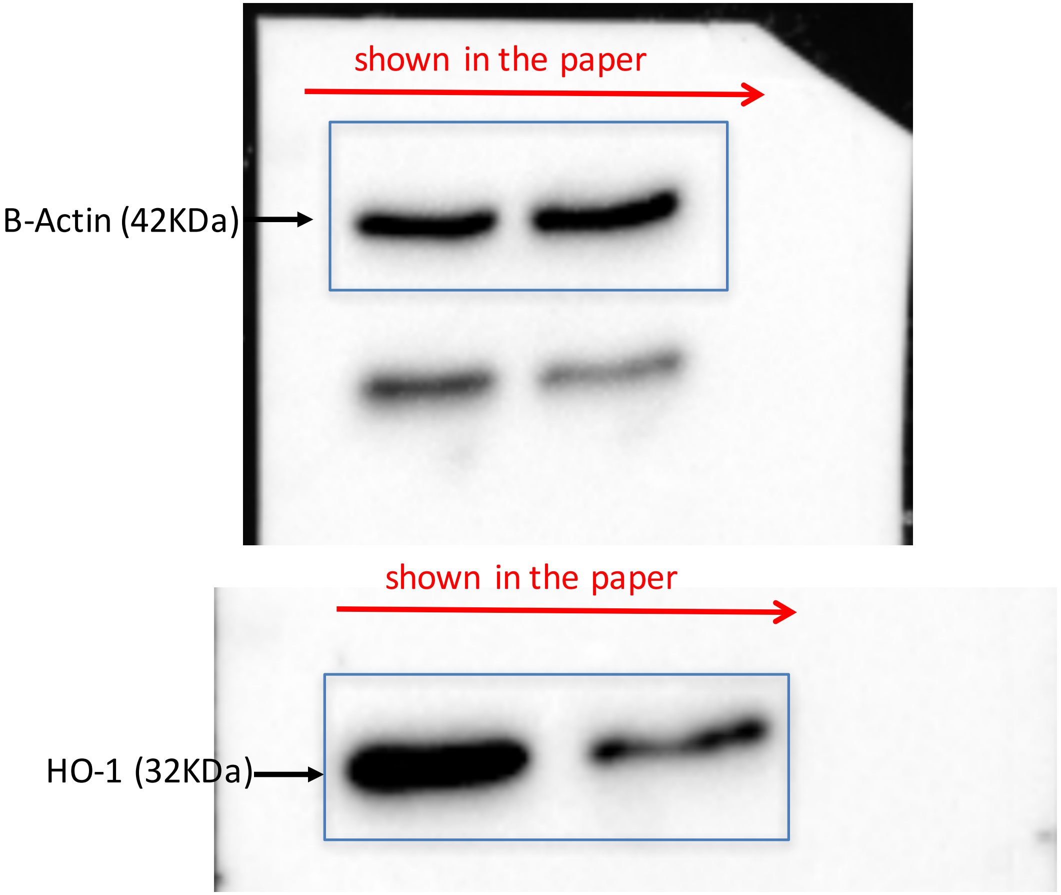

SUPPLEMENTARY FIGURE 8G
(related to Figure 3A)

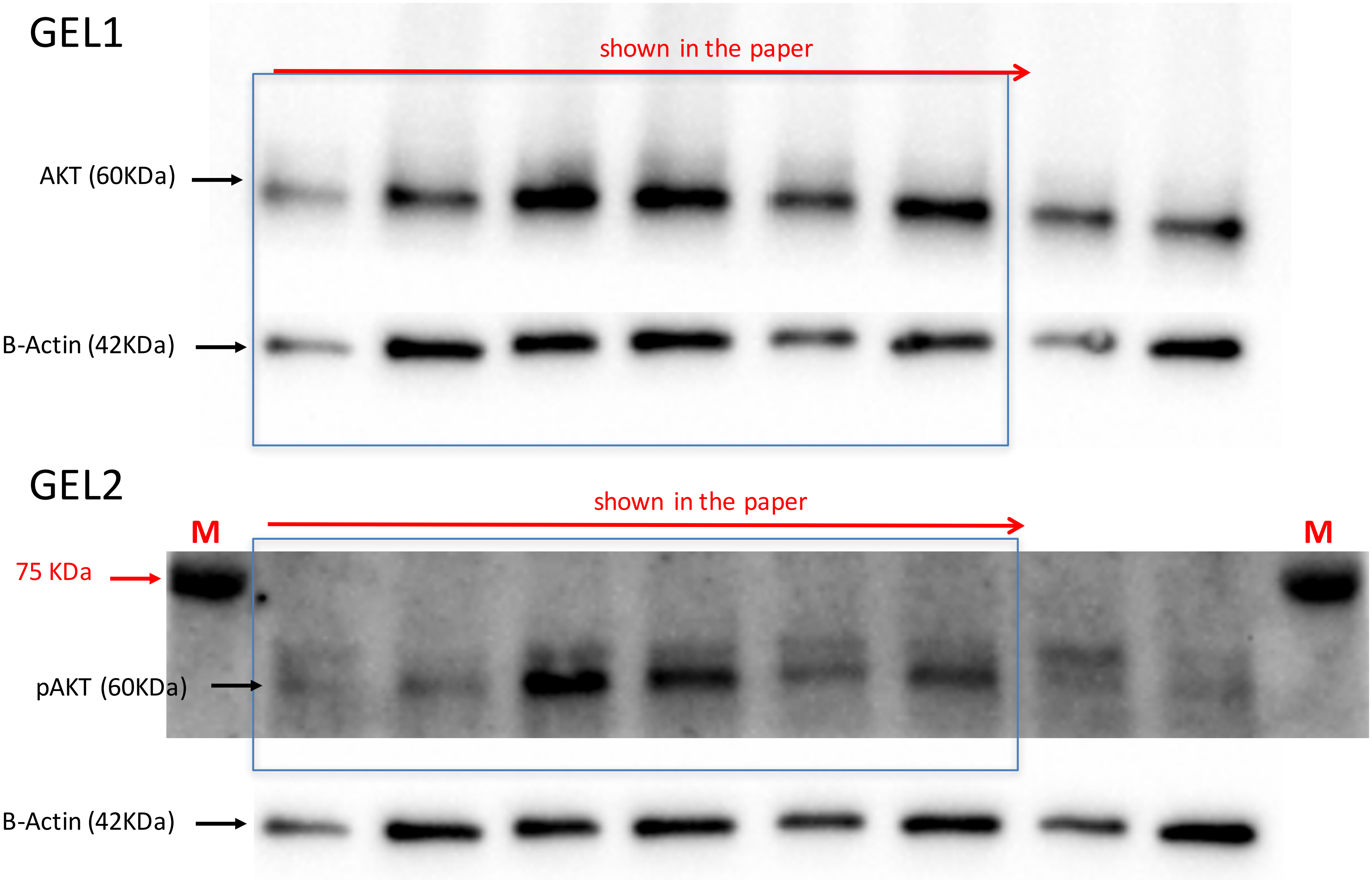

Membranes were cut before incubation with primary antibodies

SUPPLEMENTARY FIGURE 8H (related to Figure 3C)

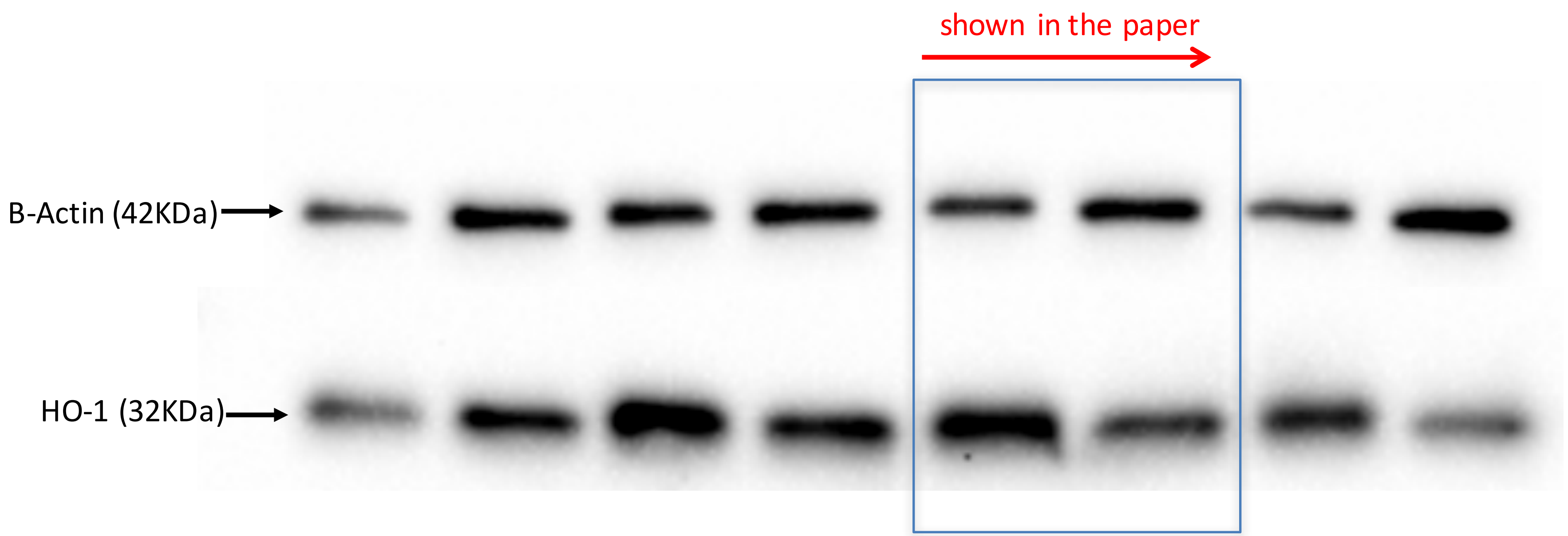

SUPPLEMENTARY FIGURE 8I (related to Figure 4B)

Membranes were cut before incubation with primary antibodies

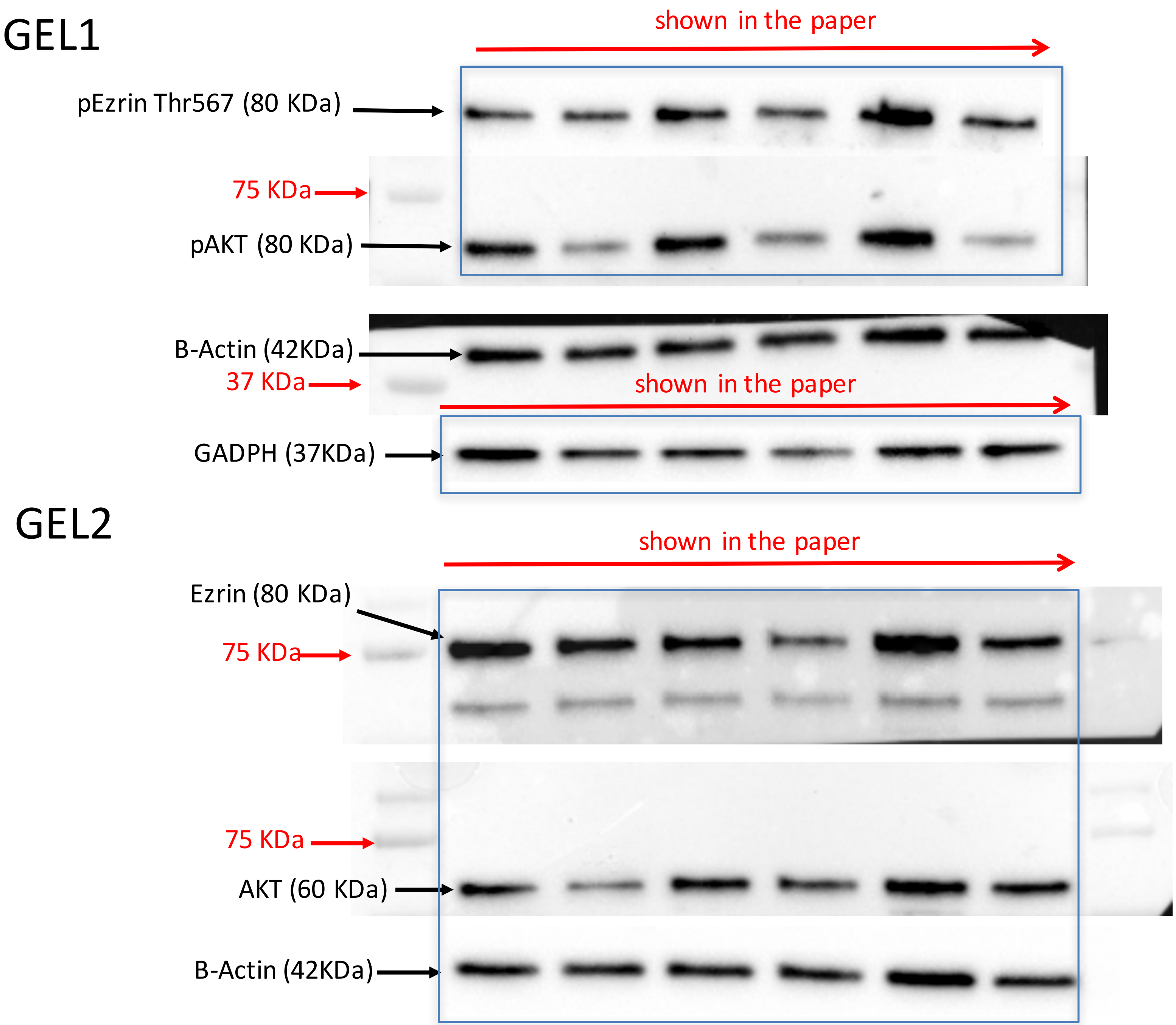

SUPPLEMENTARY FIGURE 8J
(related to Figure 4C)

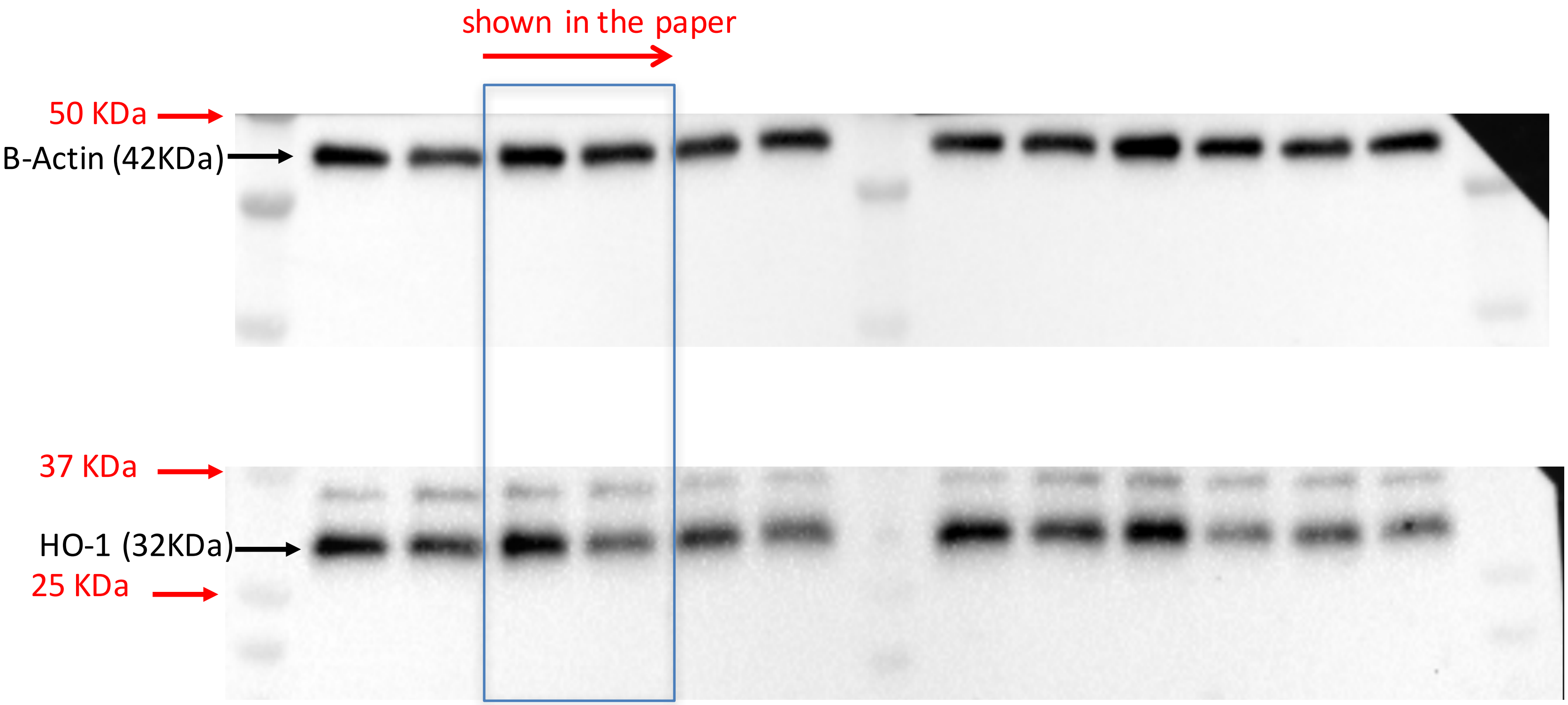

Supplement: Supplementary file 1 — Ezrin links CFTR to TLR4 signaling to orchestrate anti-bacterial immune response in macrophages [file 41598_2017_11012_MOESM1_ESM.pdf]
